# PBPK model reporting template for chemical risk assessment applications

**DOI:** 10.1016/j.yrtph.2020.104691

**Published:** 2020-06-02

**Authors:** Yu-Mei Tan, Melissa Chan, Amechi Chukwudebe, Jeanne Domoradzki, Jeffrey Fisher, C. Eric Hack, Paul Hinderliter, Kota Hirasawa, Jeremy Leonard, Annie Lumen, Alicia Paini, Hua Qian, Patricia Ruiz, John Wambaugh, Fagen Zhang, Michelle Embry

**Affiliations:** a U.S. Environmental Protection Agency, Office of Pesticide Programs, Health Effects Division, 109 TW Alexander Dr, Research Triangle Park, NC, 27709, USA; b Corteva Agriscience, Haskell R&D Center, 1090 Elkton Road, Newark, DE, 19714, USA; c BASF Corporation, 26 Davis Drive, Research Triangle Park, NC, 27709, USA; d National Center for Toxicological Research, US Food and Drug Administration, 3900 NCTR Rd, Jefferson, AR, 72079, USA; e ScitoVation, 100 Capitola Drive, Durham, NC, 27713, USA; f Syngenta Crop Protection, LLC, 410 Swing Rd, Greensboro, NC, 27409, USA; g Sumitomo Chemical Co, Ltd, 1-98, Kasugadenaka 3-chome, Konohana-ku, Osaka, 554-8558, Japan; h Oak Ridge Institute for Science and Education, 100 ORAU Way, Oak Ridge, TN, 37830, USA; i European Commission Joint Research Centre, Via E. Fermi 2749, Ispra I, 21027, Italy; j ExxonMobil Biomedical Sciences, Inc, 1545 US Hwy 22 East, Annandale, NJ, 08801, USA; k CDC-ATSDR, 4770 Buford Hwy, Mailstop S102-1, Chamblee, GA, 3034, USA; l US Environmental Protection Agency, Center for Computational Toxicology and Exposure, 109 TW Alexander Dr, Research Triangle Park, NC, 27711, USA; m The Dow Chemical Company, 1803 Building, Midland, MI, 48674, USA; n Health and Environmental Sciences Institute, 740 15th Street, NW, Suite 600, Washington, DC, 20005, USA

**Keywords:** Physiologically-based pharmacokinetic (PBPK) model, Regulatory risk assessment, Regulatory submission, Regulatory review, Reporting template, Standardized report, Harmonized report, Pharmacokinetics, Documentation

## Abstract

Physiologically-based pharmacokinetic (PBPK) modeling analysis does not stand on its own for regulatory purposes but is a robust tool to support drug/chemical safety assessment. While the development of PBPK models have grown steadily since their emergence, only a handful of models have been accepted to support regulatory purposes due to obstacles such as the lack of a standardized template for reporting PBPK analysis. Here, we expand the existing guidances designed for pharmaceutical applications by recommending additional elements that are relevant to environmental chemicals. This harmonized reporting template can be adopted and customized by public health agencies receiving PBPK model submission, and it can also serve as general guidance for submitting PBPK-related studies for publication in journals or other modeling sharing purposes. The current effort represents one of several ongoing collaborations among the PBPK modeling and risk assessment communities to promote, when appropriate, incorporating PBPK modeling to characterize the influence of pharmacokinetics on safety decisions made by regulatory agencies.

## 1. Introduction

Physiologically based pharmacokinetic (PBPK) modeling computes and predicts the concentrations of a chemical (and its metabolites) within the body over time from a given external exposure. PBPK models describe the processes of chemical absorption, distribution, metabolism, and excretion (ADME) based on physiological and biochemical mechanisms. PBPK models for pharmaceuticals and environmental chemicals have been used to predict internal chemical concentrations under conditions or in populations for which experimental data are unavailable. They can extrapolate results from preclinical to clinical trials, healthy adults to specific populations, and animals to humans (Malik et al., 2017). PBPK models can also be used to organize current knowledge on disposition mechanisms, leading to hypothesis generation and the development of follow-on studies (Abaci and Shuler, 2015; Claassen et al., 2015).

PBPK models separate physiology from drug/chemical-specific or scenario-specific (for example, dose metrics, time of exposure) characteristics, thus allowing for simulating scenarios for different subgroups within a population (Jamei, 2016). PBPK models for drugs have been used to determine doses for pediatric and adult patients, investigate drug-drug interactions, or evaluate exposure levels in patients with diseases that affect pharmacokinetics (Duan et al., 2017a, 2017b; Hsu et al., 2014; Wagner et al., 2015; Yamazaki et al., 2015). PBPK models for environmental chemicals have commonly been used to extrapolate across species, routes, life stages, and exposure duration (Andersen et al., 1987; Gentry et al., 2017; Shankaran et al., 2013; Weijs et al., 2012).

By incorporating appropriate ranges of physiological and biochemical parameter values, often through Bayesian approaches and Monte Carlo methods, PBPK models can assess implications of variability and uncertainty for a predicted outcome of interest in a population (Barton et al., 2007; Beaudouin et al., 2010; Bois et al., 2010; Fierens et al., 2016). This capability is particularly valuable when using a PBPK model to characterize genetic polymorphisms in a population (Marsousi et al., 2017; Wetmore et al., 2014). PBPK models can also assess aggregate exposure for environmental chemicals via multiple sources (for example, food, drinking water, use of consumer products) (Kenyon et al., 2016; Kim et al., 2007) and to interpret biomarker data collected from epidemiological or biomonitoring studies (Brown et al., 2015; McNally et al., 2011; Moreau et al., 2017; Tan et al., 2006; Verner et al., 2015).

Despite the many advantages offered by these applications, public health agencies often hesitate to use PBPK models to support regulatory decision-making (Chiu et al., 2007; McLanahan et al., 2012; Tan et al., 2018). Some hesitation is related to an inconsistent or non-standardized submission format to facilitate model reviews (McLanahan et al., 2012). Others hesitate to submit PBPK models because of different acceptance criteria among agencies and countries (Paini et al., 2017). A harmonized template for reporting PBPK analyses to regulatory agencies does not currently exist. Most model developers adhere to the same established best practices for characterizing and applying PBPK analyses (USEPA, 2006; WHO, 2010), but the format and content of reports on these analyses can vary significantly because individual investigators subjectively determine the most critical elements to report.

Harmonizing a PBPK reporting template among global public health agencies should reduce the burden of preparing different reports for individual agencies when the modeling analysis is the same. Reviewers can also benefit, since interpreting modeling analyses in various contexts and formats is time-consuming especially when reviewers have limited expertise in PBPK modeling (Tan et al., 2018). Recognizing that a standardized reporting template can facilitate efficient assessment, consistent application, and timely decision-making during regulatory review, the U.S. Food and Drug Administration (FDA) recently published a guidance for reporting PBPK analyses in regulatory submissions (USFDA, 2018). The guidance does not recommend best practices for conducting PBPK analyses or evaluating PBPK approaches for regulatory applications; rather, it outlines standardized content and format for PBPK study reports submitted to the FDA (USFDA, 2018). The European Medicines Agency (EMA) published similar guidelines for reporting PBPK modeling and simulation as part of regulatory submissions (EMA, 2019). The EMA guidance clarifies their expectation on qualifying a PBPK platform for the intended use.

The purpose of the PBPK reporting template presented in this article is to expand on the existing guidance designed for pharmaceutical applications (EMA, 2019; Shebley et al., 2018; USFDA, 2018). Table 1 provides an overview of how this new reporting template compares to these and other existing templates or guidance documents. We recommend additional elements so that the reporting template can also be used by agencies assessing the safety of environmental chemicals. An example is presented in the Supplemental Data File to demonstrate the use of this template to report an PBPK analysis on estragole.

This template can be adopted and customized by regulatory agencies receiving submissions using PBPK modeling. It can facilitate communication between model developers and reviewers/users, since modeling processes and outputs are organized in a structure familiar to both parties. It can serve as general guidance for submitting PBPK-related studies for other purposes, for example, publishing study results in journals or sharing modeling results with collaborators. It can also be used as part of a training package for both reviews and new modelers, since the recommended content is based on key principles in other guidance documents (Caldwell et al., 2012; Clark et al., 2004; USEPA, 2006; WHO, 2010). Since these other guidance documents already address good practices in developing, applying and reviewing PBPK analysis (EFSA, 2014; USEPA, 2006; WHO, 2010) these topics will not be reiterated in the current article.

## 2. Template elements

### 2.1. Executive summary

In this section, clearly convey how the PBPK analysis addresses a specific scientific question in support of a regulatory decision. Including the following information:

- The rationale and intended regulatory purpose for conducting the PBPK analysis
- An overview of the model and its applications
- A summary of key conclusions

### 2.2. Background information

In this section, provide sufficient background about the chemical of interest to place the PBPK modeling analysis in the context of regulatory application, which can include the following information:

- A high-level synopsis of a chemical’s physicochemical, pharmacokinetic or toxicokinetic, and/or pharmacodynamic/or toxicodynamic properties relevant for the PBPK model and its application
- A brief summary of known exposure and toxicity for an environmental chemical, or known dose, toxicity, and efficacy of a drug
- A brief regulatory history to provide context for conducting the analysis
- A history of previous PBPK models and/or reports on the same chemical submitted for different applications or different agencies, or previously published in open literature; in some cases, models for isomers or homologs of chemicals can be used as surrogates
- A summary of relevant data used for model calibration, such as dose-response or exposure-response data, time-concentration data in plasma, target tissue/organs, or surrogates of target tissue/organs
- A summary of available data for model evaluation, such as time-concentration data measured in test species or from clinical trials or biomonitoring studies

### 2.3. Model purpose

In this section, clearly articulate the regulatory question(s) the PBPK model approach addresses, such as through a problem formulation or fit-for-purpose statement.

### 2.4. Materials and methods

In this section, provide a detailed description of the protocol and procedures to follow in developing the model and completing modeling analysis. Include enough information for reviewers to evaluate the quality and accuracy of the analysis, replicate the analysis, or conduct additional simulations using the model when necessary. The section can include the following seven components.

#### 2.4.1. Modeling strategy

In this section, include a well-thought-out modeling procedure from data preparation to model development, model verification/evaluation, and model applications (Sager et al., 2015; WHO, 2010; Zhao et al., 2011). The procedure should follow the path of a workflow, decision tree, roadmap, or other representation. In this section, also demonstrate the relevance of the modeling strategy to the modeling purpose.

#### 2.4.2. Summary of data for model development and evaluation

In this section, include the available data (with their sources) used to inform the model structure, calibrate model parameters, and evaluate the predictive capability of the model. Details of the key studies used to obtain these data need to be clearly described in this section or submitted as supplemental materials, such as cell type or subcellular fraction used, preparation procedures, substrate concentrations, incubation time, or other experimental conditions necessary to repeat the measurements. Some examples of information to be included are listed below:

- Relevant chemical(s) information, such as physicochemical properties and relevant exposure routes
- Toxicity studies that determine mode of action and toxic moiety (for example, parent and/or metabolites)
- Mass balance studies using a radiolabeled chemical
- Metabolism studies to determine metabolic pathways and rates of metabolism
- Time-concentration data measured in plasma, organs/tissues, or excreta
- Biochemical parameters measured using *in vitro* systems or predicted using *in silico* tools, such as intrinsic clearances of enzymes in an organ/tissue, kinetic constants for an enzyme, or binding constants

#### 2.4.3. Model development and structure

**Fig. 1.**
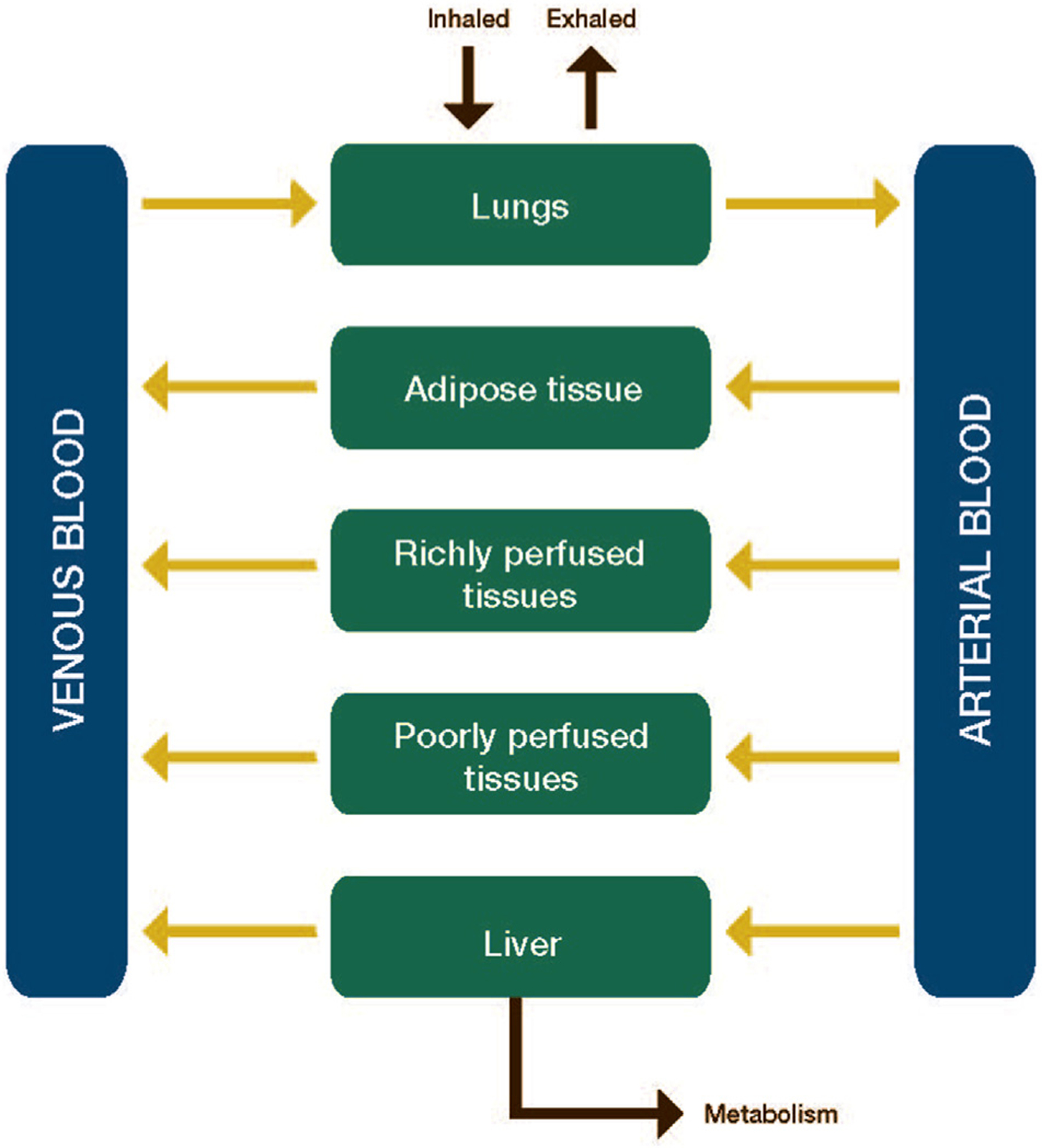
General schematic diagram of a physiologically based pharmacokinetic (PBPK) model.

In this section, include key assumptions (for example, population of interest, perfusion- or diffusion-limited compartments) used to determine the model structure. If a generic PBPK modeling platform is used, provide relevant references or a users’ manual that clearly describes the model structure. We recommend a schematic diagram to present the PBPK model structure (Fig. 1). In addition, explain whether the PBPK model replicates a published model, is refined/modified from an existing model, or is a new model. Include clear descriptions and justifications for any customized changes made to any previous model.

#### 2.4.4. Model equations

In addition to all electronic files containing model code, include mathematical equations for the PBPK model to allow for replication in other software packages or programming languages when needed.

- Ensure that parameter names used in the equations are consistent with those coded in the software program.
- If a commercial PBPK software program is used to build and run a PBPK model, providing model equations may not be necessary. The software, however, must be qualified to ensure that it does what it is intended to do from a computational perspective, and that guidance on software qualification can be found in other documents (EMA, 2019; Shebley et al., 2018).

#### 2.4.5. Model parameters

Report parameters in both tabular and text format.

- At a minimum, include in the table parameter names/symbols, meanings, values, units, and sources of parameter values.
- If a parameter has more than one value from various sources, justify the chosen or estimated value (for example, average of values obtained from various sources).
- Clearly identify allometric scaling from the units reported in the parameter table (for example, L/h/kg^0.75^). Sources of parameter values include literature/reports, measurements using *in vitro* or *in vivo* methods, predictions using *in silico* methods (for example, quantitative structure-activity relationship), databases embedded in commercial PBPK software, and estimations from fitting model predictions to observed data (see section 4.6 below).
- For literature sources, include enough details for easy query to methods used to measure or predict a parameter value or to verify that a parameter value is the same as reported in the literature.
- For a published article, provide a full citation or unique identifier (for example, PubMed PMID, article DOI) along with page numbers and/or table/figure numbers, or cite well-known and well-respected articles, such as (Brown et al., 1997; ICRP, 1975; ICRP, 2002). Cite the original source for a parameter value, rather than a secondary source that references other publications.
- If ancillary studies are conducted to measure or predict parameter values, submit unpublished reports describing these studies as supplemental materials.

Commercial PBPK software programs usually include embedded databases for physiological parameter values. They also integrate built-in algorithms to estimate chemical-specific parameter values.

- Export parameter values to files (for example, text file or spreadsheet) when possible. Include these files as supplemental materials and provide the rationale for choosing the default values in the report.
- For parameter values that cannot be exported, either provide references describing the sources of default values or create a table summarizing model parameters as described above.
- To justify modifications and describe sources of new values, include references for any modification to the default values in the main report.

In many instances, literature values are not available for all model parameters. Describe the details of approaches and tools used to determine values of parameters in this section or submitted as supplemental materials. One example is the mathematical or table functions for describing age-dependent changes in physiology. If such mathematical functions are obtained by fitting to a series of data points, include references from which raw data are obtained, along with a description of curve fitting analysis, regression procedures, or other methods used to construct these functions.

Document optimization of model parameters.

- When tissue or plasma/blood concentration data are used to optimize model parameters, document both the optimized parameters and optimized aspects of model performance (for example, peak concentration in plasma, root mean square error for time-concentration data in the target tissue).
- When visual inspection is used for model optimization, state and justify the optimization threshold (for example, parameters are manually adjusted to yield predictions within a specific fold of observations).
- When formal statistical methods are used for model optimization, provide and reference any data sources and details (for example, descriptions of priors for Bayesian analysis).
- When sequential or iterative processes are involved in model optimization, provide details on those processes (for example, fitting one parameter with one dataset before fitting another parameter with another dataset).

Document evaluation of model performance by comparing model predictions to datasets not involved in the optimization process, as well as sensitivity analysis that is helpful in assessing the identifiability of optimized parameters, in section 5 (“Modeling results”).

When submitting a probabilistic PBPK model that accounts for variability and uncertainty of parameter values, a separate parameter table may be needed in addition to the parameter table prepared for the deterministic model.

- List parameter names/symbols, meanings, types of probability distributions (for example, normal, log-normal, triangular), parameters for describing probability distributions (for example, mean, variance, truncated range), units, and sources of parameter values.
- Describe any modifications made to the deterministic model to maintain mass balance and physiological plausibility.
- Clarify whether parameter uncertainty and variability are considered together or separately. Although separation of parameter uncertainty and variability is theoretically possible using hierarchical, population-based models, data are typically inadequate to achieve such a level or granularity.

#### 2.4.6. Model simulations

In this section, provide the following details of the simulation conditions for model development, evaluation, and applications.

- Exposure/administration characteristics, such as routes (for example, inhalation, oral intake, ocular administration, skin contact), time at which each exposure/administration occurs and duration/length of exposure/administration (for example, 4 h per day starting at 7 a.m. for 2 weeks), formulation (for example, powder, solution, suspension, vehicle/solvent) or other physical properties (for example, particle size, surface area), and feeding or fasting condition
- Doses/concentrations of chemical administered/exposed, and any information required to determine the apparent doses such as ventilation rates
- Types of samples (for example, radiolabeled compound, parent chemical in plasma, metabolites in urine) and time of sample collection
- Characteristics of the test subjects and the simulated virtual population, such as species, number of subjects, gender, ethnicity, life stage, healthy vs. disease state, relaxed vs. active state
- For pharmaceuticals, number of simulations for a specific administration scenario and number of virtual subjects in each simulation trial
- For probabilistic simulations, methods for analysis (for example, Monte Carlo, Markov Chain Monte Carlo), number of iterations for a specific dosing/exposure scenario, and associated data involved in the analysis (for example, observational data used to update the priors in Markov Chain Monte Carlo analysis)

#### 2.4.7. Software

In this section, include the following elements:

- Name and version of the software
- Link to the software product website and, if necessary, the user’s manual
- Specification of the ordinary differential equation solvers, optimization, and statistical algorithms
- Step-by-step instructions on how to run the model simulations for all scenarios presented in the report

### 2.5. Modeling results

In this section, demonstrate and discuss the predictive capability and robustness of the PBPK model for the intended purposes. The section may include the following three components:

- Comparison of model predictions with available data
- Analysis of parameter sensitivity and variability, and model uncertainty for any scenarios in which the model is used
- Model applicability

#### 2.5.1. Model evaluation

In this section, present results that demonstrate the capability of the model to simulate available *in vivo* time-concentration data in figures or tables and summarize results in the text.

- Clearly distinguish the results showing the model fit to training dataset, and the results showing the model’s ability to simulate other datasets.
- Describe statistical tests or other approaches (for example, visual observation) used to investigate the goodness-of-fit for deterministic and probabilistic simulations (as applicable) in conjunction with the interpretation of test results.
- Discuss model performance in the context of the intended applications.

Graphical comparisons between the observed data and model predictions are presented in two common ways.

**Fig. 2.**
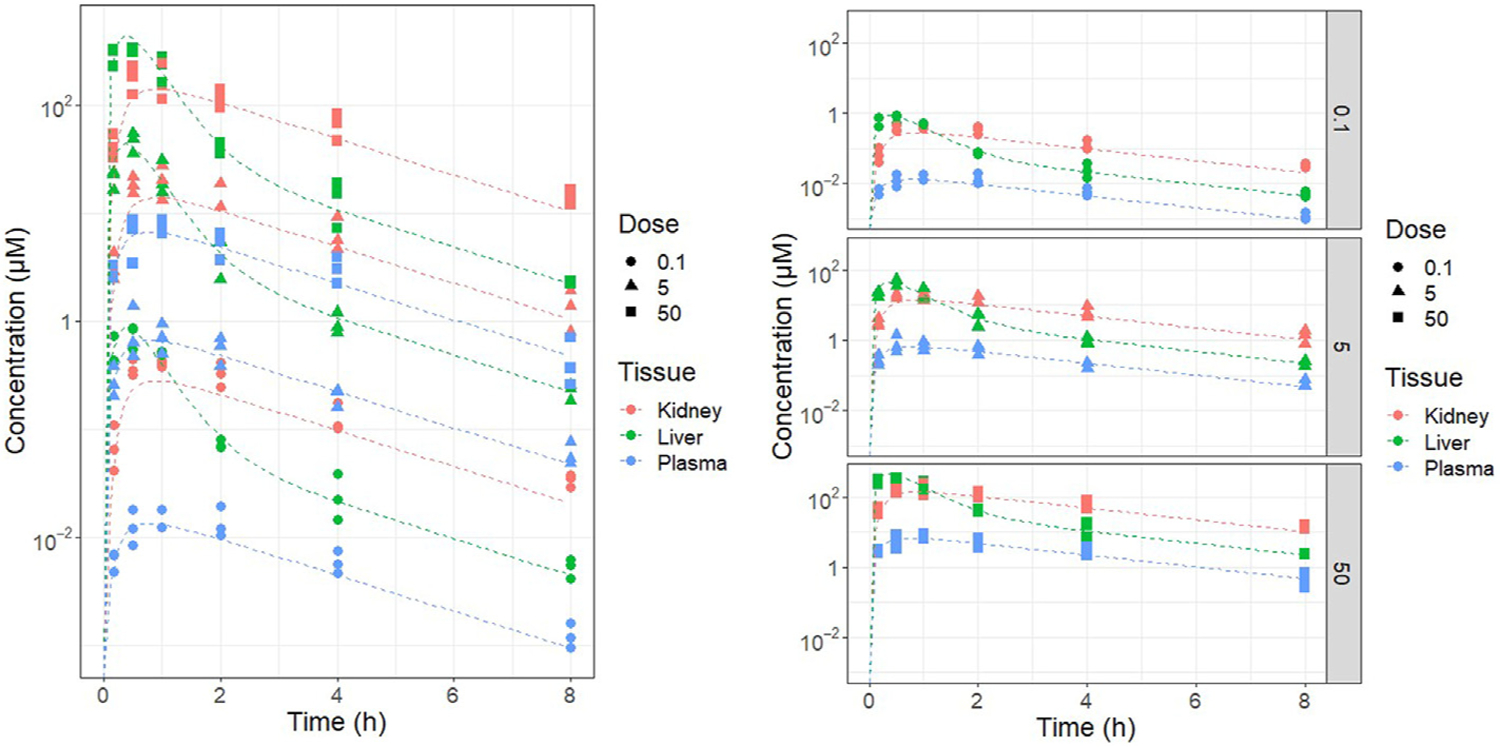
Comparison between predicted vs. observed concentrations over time.

**Fig. 3.**
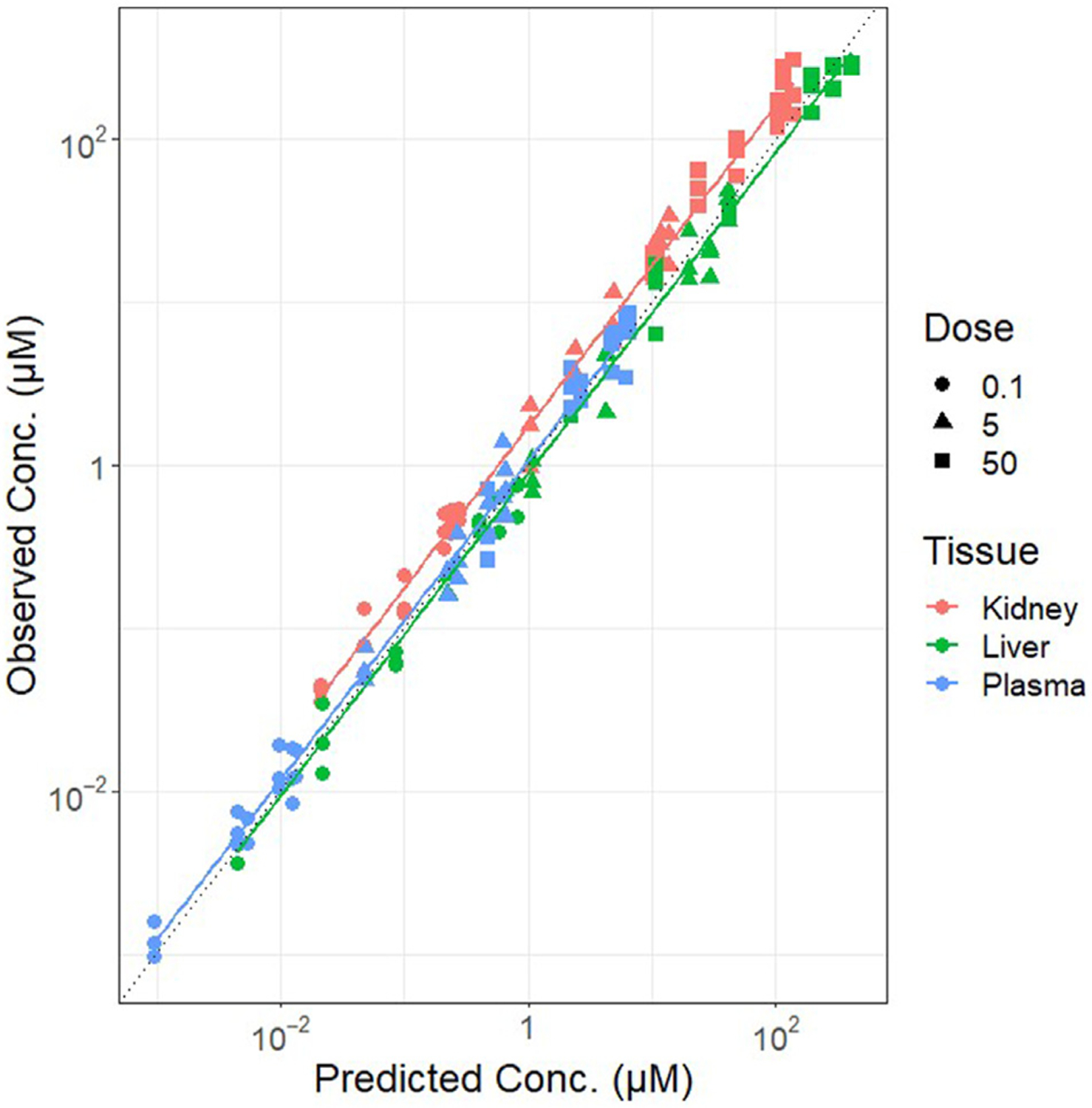
Observation vs. prediction plot for quantitative analysis of predictive ability.

- A concentration vs. time plot provides a qualitative evaluation of model predictions (for example, Fig. 2).
- An observed vs. predicted outputs plot can provide a quantitative evaluation of model predictions, typically with the results of a linear regression of the observed vs. predicted values (for example, Fig. 3) along with summary statistics such as coefficient of determination (R^2^). The dotted line in Fig. 3 indicates the “identity” line along which perfect predictions will lie. This type of plot can be used to detect model biases and the overall predictive ability of the model.

#### 2.5.2. Sensitivity, uncertainty, and variability analyses

Model reliability is critical for regulatory acceptance; key components are parameter sensitivity, uncertainty, and variability analyses. Sensitivity analysis that is conducted with respect to parameter identifiability may be structural or statistical (Cobelli and DiStefano, 1980) and can be local (considering one parameter at a time) or global (considering all parameters jointly) (McNally et al., 2011). Structural identifiability refers to the level of influence that a parameter exerts on the model output. Statistical identifiability refers to the ability of the available data to inform the value of a parameter (Bernillon and Bois, 2000). It may involve Bayesian analysis (Garcia et al., 2015). Clearly note model parameters that are not identifiable from fitting model predictions to available data; they may affect the model’s ability to extrapolate to new conditions (Chiu et al., 2007). Report the following information:

- Model outputs for which sensitivity analysis is conducted and rationale for the choice of model outputs in the context of the intended applications
- The type of sensitivity analysis (for example, local vs. global)
- The sampling approach (for example, one at a time vs. all at a time, various time points vs. at steady state)
- The sampling methods, such as eFAST (Marino et al., 2008), Morris method (Morris, 1991), sensitivity coefficient calculation (e.g., absolute change ratio or percentage change ratio)
- An overview of key parameters identified as sensitivity parameters
- A statement of overall impact on model outputs and overall reliability.

In addition to sensitivity analysis, document approaches and results from uncertainty and variability analyses in this section. *Uncertainty* refers to lack of knowledge about the true value of model parameters and can be reduced through additional observation. *Variability* describes how a parameter value changes across a population of individuals, arising from differences in life stages, genetics, behavior, or activity. It cannot be reduced through further observations, although it can be more precisely characterized. In addition to model robustness, analysis of these model properties can inform model applicability, limitations, and potential refinement.

Since uncertainty, variability, and global sensitivity analyses are commonly conducted for a subset of the model parameters, identify the parameters in the analyses and provide criteria for inclusion/exclusion. Concepts and interpretations of these analyses are intertwined; hence, simultaneously consider the degree of sensitivity with uncertainty/variability of model parameters for meaningful assessment of model reliability.

- Parameters that are uncertain and have substantial impact on model output reduce model robustness and reliability.
- Uncertainty and variability of parameters to which the model has little sensitivity may be of negligible concern.
- For parameters with precisely known values (that is, low uncertainty), sensitivity may still be of interest where population variability is high.

**Fig. 4.**
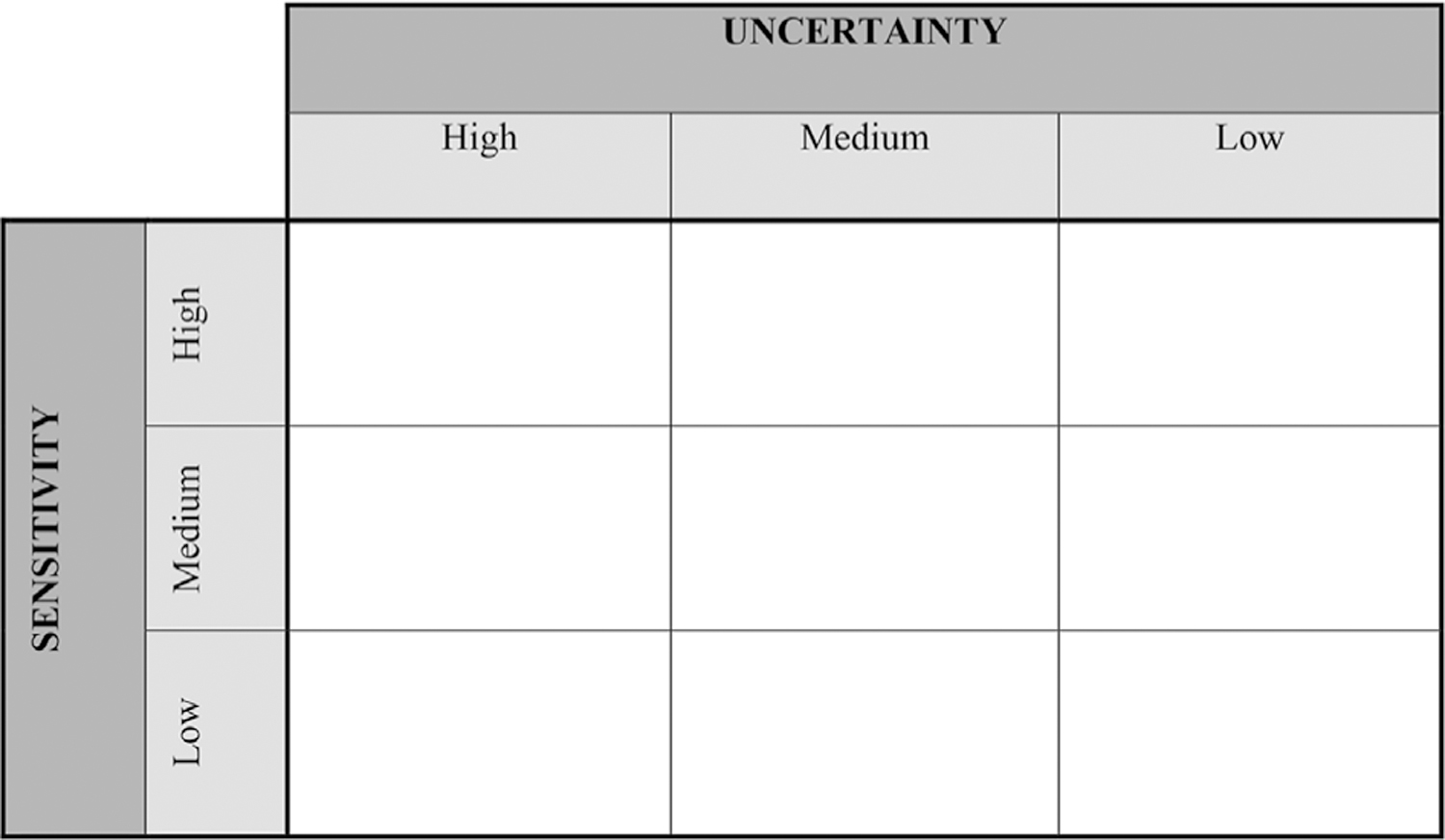
Adapted from (Meek et al., 2013; WHO, 2010).

Therefore, to facilitate model evaluation, present the results of these analyses together. For example, use a graphical representation to illustrate uncertainty versus sensitivity in low, medium, and high categories (Fig. 4, adopted from (Meek et al., 2013). Clearly state the definition of low, medium, and high uncertainty and sensitivity, since these levels can be necessarily subjective.

#### 2.5.3. Model applicability

In this section, present how the PBPK model is used, including modeling results for its intended applications (for example, to derive an interspecies extrapolation factor). Summarize the levels of confidence or uncertainty associated with the intended application. For example, these levels may be based on the robustness of the model in simulating the underlying biological mechanisms, or knowledge and data used to support major assumptions in modeling analysis. Clarify model limitations and domains of applicability.

### 2.6. Discussion and conclusions

In this section, briefly reiterate the following information:

- The question to be answered or hypothesis to be tested
- An explanation of why a PBPK model is required or preferred
- A summary of how the PBPK model helps address the question
- A summary of modeling analysis (for example, a rat and human model for the chemical with oral and inhalation routes of exposure)
- A highlight of key results and interpretation of these results (for example, magnitude of difference between rat and human dose metrics, or linear or saturable kinetics at doses up to some level)
- A discussion of the uncertainties and limitations of the model (for example, sensitive parameters that are assumed to be the same across species but without data to confirm)

If PBPK modeling analysis is one piece of evidence addressing the questions, include other pieces of evidence in the discussion.

### 2.7. Electronic files and supporting documents

Provide electronic files and supporting documents with the main report. Examples of electronic files include model code, metadata, parameter tables, and simulation inputs/outputs. Examples of supporting documents include reports summarizing *in vivo* studies that provide the time-concentration data for model calibration or evaluation, or *in vitro* studies that measure chemical absorption or metabolism.

- Include all materials related to the modeling history, and justification of modeling strategies, model parameterization, and model evaluation/verification/qualification, as well as model equations, if they are too long to be included in the main report.
- Provide references that contain model equations or approaches for obtaining default parameter values, as well as user manuals for commercial PBPK software programs.
- Provide a document with step-by-step instructions for running the model and generating tables/figures found in the main report.
- When possible, documentation quality practices followed by the model developer’s organization, and/or third-party review of the modeling analysis.

### 2.8. Appendices

In this section, include a table of acronyms and abbreviations, a list of tables, a list of figures, and references. When possible, use hyperlinks in the main report to cross-reference tables, figures, and references.

## Discussion & conclusions

This harmonized PBPK reporting template can be used in various ways, including providing clear guidance on critical elements evaluated during regulatory review processes. The template will also reduce the burden on investigators who may otherwise prepare separate reports for various regulatory jurisdictions or agencies, using the identical modeling analysis. Adapted from existing guidance templates (for example, USFDA, 2018; EMA, 2019), this template recommends additional elements when using PBPK models to evaluate the safety of environmental chemicals. Therefore, this general template can be applied to submitting models for both environmental chemicals and pharmaceuticals. This template is also a good starting point for the development of a new OECD Harmonized Template (OHT) (http://www.oecd.org/ehs/templates) to report PBPK models for chemical risk assessment and regulatory uses.

This template has additional contexts of use beyond the regulatory sphere, including submitting and sharing PBPK-related studies (for example, for peer-reviewed publication or scientific collaborations), ensuring transparent communication between reviewers and model developers, and training for new modelers and decision makers. Using this template will help standardize PBPK model reporting and communication and thereby enhance their application and regulatory acceptance.

## Supplementary Material

Suppl Info

## Figures and Tables

**Table 1:** Summary of the elements included in this template (Tan et al., 2020) compared to other existing templates and guidance documents.

	Current paper (Tan et al., 2020)	WHO, 2010,^a^	USFDA, 2018	EMA, 2019
**Executive Summary Background/Introduction**	Included • Chemical’s physicochemical, PK and PD properties • Known exposure, toxicity, efficacy • PBPK-related regulatory history • Cross-referencing other PBPK efforts • Relevant data used for model calibration • Relevant data used for model evaluation	N/A • Critical effect • PK • Mode of action/relevant dose metric • Scope for model application	Included • Drug’s physicochemical, PK and PD properties • Exposure-response relationship for efficacy and safety • PBPK-related regulatory history • Cross-referencing previously submitted PBPK reports	N/A • Drug’s physiochemical properties • Drug’s pharmacokinetic parameters • Clinical studies • Data related to the intended purpose • A quantitative mass-balance diagram presenting elimination pathways • Dose- or time-dependent PK, drug-drug interactions, pharmacogenetic differences • Exposure-response relationship for efficacy and safety
**Model Purpose** **Materials & Methods**	Included • Modeling strategy • Summary of data for model development and evaluation • Model development and structure • Model equations • Model parameters • Model simulations • Software	In “Introduction” PBPK model: characterization and evaluation • Model capability and selection • Model structure and biological characterization • Parameter estimation and analysis • Purpose-specific model evaluation • Model documentation • Model peer review	In “Executive Summary” • Modeling strategy • Modeling parameters • Simulation design • Electronic files and other documentation • Software	In “Objective and Regulatory Purpose” Model parameters • Assumptions • System-dependent parameters • Drug parameters and the drug model Model development Simulation of the intended scenario
**Results**	• Model evaluation • Sensitivity, uncertainty, and variability analyses • Model applicability	PBPK modeling and evaluation of dose metrics PBPK model application and comparison with default	• Model verification and modification • Model application	Platform and drug model evaluation • Sensitivity analyses • Evaluation of the predictive performance of the drug model Results
**Discussion & Conclusions**	Included	N/A	Included	Discussion of the regulatory application
**Electronic files and Supporting Documents**	Included	N/A	In “Materials and Methods”	Qualification of the PBPK platform
**Appendices**	Included	N/A	Included	N/A

a This document (WHO, 2010) is not intended to be used as a template for regulatory submission purposes.

## Abbreviations

ADME: absorption, distribution, metabolism, and excretion
DOI: digital object identifiers
eFAST: Extended Fourier Amplitude Sensitivity Test
EMA: European Medicines Agency
HESI: Health and Environmental Sciences Institute
OECD: Organization for Economic Co-operation Development
PBPK: Physiologically Based Pharmacokinetic
PMID: PubMed Identification
USEPA: U.S. Environmental Protection Agency
USFDA: U.S. Food and Drug Administration
WHO: World Health Organization
