# Supplementary material for: PBPK model reporting template for chemical risk assessment applications
Source: Regul Toxicol Pharmacol. Author manuscript; Available in PMC 2021 Aug 1. (PMC8188465; doi:10.1016/j.yrtph.2020.104691)
Supplement: Suppl Info [file NIHMS1700380-supplement-Suppl_Info.docx]

**Appendix**

**PBPK model reporting template for chemical risk assessment applications: Example for estragole DNA adduct formation**

*Note: The example was prepared to illustrate how the PBPK model reporting template could be used, using estragole DNA adduct formation as an example. The authors thank Dr. Alicia Paini (European Commission Joint Research Centre) for preparing this example.*

**Template elements**

# Table of content

[Table of content 2](#_Toc30515963)

[1. Executive summary 3](#_Toc30515964)

[2. Background information 3](#_Toc30515965)

[3. Model purpose 8](#_Toc30515966)

[4. Materials and methods 8](#_Toc30515967)

[4.1. Modeling strategy 9](#_Toc30515968)

[4.2. Summary of data for model development and evaluation 10](#_Toc30515969)

[4.3. Model development and structure 12](#_Toc30515970)

[4.4. Model equations 13](#_Toc30515971)

[4.5. Model parameters 15](#_Toc30515972)

[4.6. Model simulations 18](#_Toc30515973)

[4.7. Software 18](#_Toc30515974)

[Step-by-step instructions: 18](#_Toc30515975)

[5. Modeling results 18](#_Toc30515976)

[5.1. Model evaluation 18](#_Toc30515977)

[5.2. Sensitivity, uncertainty, and variability analyses 23](#_Toc30515978)

[5.3. Model applicability 25](#_Toc30515979)

[6. Discussion and conclusions 26](#_Toc30515980)

[7. Electronic files and supporting documents 27](#_Toc30515981)

[Estragole rat PBPK model Model Code 27](#_Toc30515982)

[8. Appendices 33](#_Toc30515983)

[Abbreviations 33](#_Toc30515984)

[References 34](#_Toc30515985)

# 1. Executive summary

The present PBPK model was built to predict estragole DNA adduct formation, taking the kinetic modelling one step closer to the ultimate endpoint of tumor formation, as part of a new strategy for low dose cancer risk assessment. At the present state-of-the-art there is no scientific consensus on how to perform the risk assessment of these compounds when present at low levels in a complex food matrix. In order to refine the evaluation of the risks associated with these food-borne genotoxic carcinogens, information on their mode of action (MOA) at low versus high doses, on species differences in toxicokinetics and toxicodynamics, including dose- and species- dependent occurrence of DNA damage and repair, and on effects on expression of relevant enzymes, is required. The development of a PBPK model predicting the in vivo dose dependent DNA adduct formation in rat liver based on in vitro data only. The PBPK model allows prediction of the levels of 1′-hydroxyestragole and 1′-sulfooxyestragole in the liver of rat and an in vitro concentration response curve for DNA adduct formation in cultured rat primary hepatocytes exposed to 1′- hydroxyestragole. The PBPK model was validated and evaluated by quantifying the dose-dependent estragole DNA adduct formation in Sprague Dawley (SD) rat liver and measuring the urinary excretion of 1′- hydroxyestragole glucuronide. The model allows prediction of dose dependent bioactivation, detoxification, and DNA adduct formation of estragole in rats. The PBPK model described herein is based on the following articles by Punt et al., (2008) and Paini et al., (2010, 2012).

***Version Estragole_V1_2020***

***Time to compile around 12 hrs***

# 2. Background information

The purpose of development of this PBPK model was to predict estragole kinetics and formation of DNA adducts. Such models will facilitate risk assessment because will facilitate extrapolation from high to low dose levels, and in a next stage between species (rat-human) and between individuals.

**Estragole (C10H12O, CAS 140-67-0; MW 148.2 g/mol; LogKow = 3.47),** is an alkenylbenzene, the chemical structure consists of a benzene ring substituted with a methoxy group and a propenyl group (Figure 1).


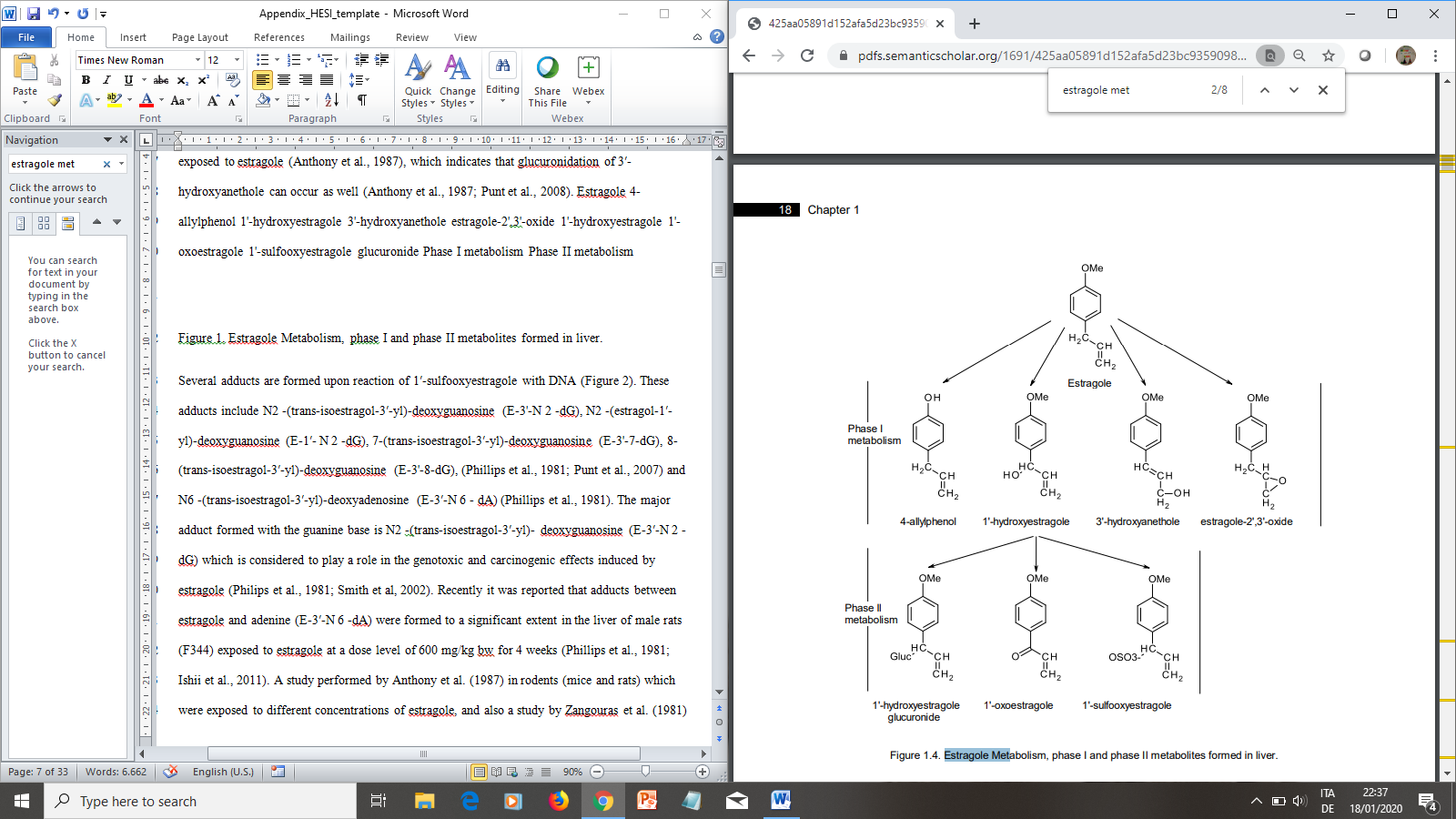


Figure 1. Estragole Metabolism, phase I and phase II metabolites formed in liver.

Estragole is a natural ingredient of several herbs and spices such as: basil, nutmeg, fennel, marjoram, tarragon and anise, and these provide one of the sources of human exposure. Since estragole containing extracts are used in food processing, exposure to this compound can also occur in food products containing the extracts or in food products containing the spices and herbs. Basil, has amount of estragole in a range of 0.05 – 19.30 mg/kg (Siano et al., 2003). Basil-based food supplements contain from 0.20 up to 241.56 mg estragole/g basil food supplement (Van den Berg et al., 2011). Estragole was found to be carcinogenic when administered to rodents at high dose levels. However, when used at low dose levels as a flavor, estragole was considered to be generally recognized as safe (GRAS) in 1965 by the Expert Panel of the Flavour and Extract Manufacturer’s Association (FEMA). Estragole was approved by the US food and Drug Administration (FDA) for food use as a flavor. In 2002 the FEMA Expert Panel reviewed the safety of the use of estragole as a flavor and estimated that the average intake from natural flavoring complexes is approximately 0.01 mg/kg bw/day (Smith et al., 2002). Estragole has also been evaluated by the European Scientific Committee on Food, with the final opinion pointing to the genotoxic and carcinogenic properties, and suggesting restrictions in its use in food (SCF, 2001). In the evaluation of the European Committee on Food the estimated intake of estragole, calculated based on its proposed uses and use levels in various food categories, amounted to 0.07 mg/kg bw/day estragole (SCF, 2001).

**Estragole Mode of Action:** Upon oral intake, estragole undergoes rapid and essentially complete absorption along the gastrointestinal tract (Anthony et al., 1987). Abdo et al. reported that estragole could be already adsorbed in the stomach (Abdo et al., 2001). Estragole itself is not reactive but upon its absorption in the gastrointestinal track the compound is transported to the liver, where it can be either bioactivated or detoxified (Smith et al., 2002). Figure 1 presents an overview of the various phase I and phase II metabolites produced resulting in bioactivation or detoxification of estragole. Estragole is detoxified via O-demethylation to 4-allylphenol and via epoxidation to estragole 2,3-oxide (Phillips et al., 1981; Smith et al., 2002). The main pathway for bioactivation of estragole proceeds by hydroxylation on the allyl side chain by cytochrome P450 enzymes resulting in the formation of **1′-hydroxyestragole** (Swanson et al., 1981), **(C_10_H_12_O_2_ ;CAS 51410-44-7; 164.2 g/mol; Log Kow (KOWWIN v1.67 estimate) = 1.93).** Subsequent sulfonation of 1′- hydroxyestragole by sulfotransferases gives rise to formation of the unstable ultimate carcinogenic metabolite 1′-sulfooxyestragole which decomposes to generate the reactive carbocation which covalently binds to DNA and protein (Phillips et al., 1981).

1′-Hydroxyestragole can be detoxified by glucuronidation or via oxidation to 1′-oxoestragole (Bock et al., 1978; Iyer et al., 2003; Phillips et al., 1981). Cytochrome P450 mediated oxidation of the allyl side chain of estragole may also result in formation of 3′-hydroxyanethole that can be oxidized to 4-methoxycinnamic acid which in turn can further be oxidized to 4-methoxybenzoic acid. Glucuronosyl conjugates of 3′-hydroxyanethole have also been observed in urine of rats exposed to estragole (Anthony et al., 1987), which indicates that glucuronidation of 3′-hydroxyanethole can occur as well (Anthony et al., 1987; Punt et al., 2008). Several adducts are formed upon reaction of 1′-sulfooxyestragole with DNA (Figure 2). These adducts include N2 -(trans-isoestragol-3′-yl)-deoxyguanosine (E-3'-N 2 -dG), N2 -(estragol-1′-yl)-deoxyguanosine (E-1′- N 2 -dG), 7-(trans-isoestragol-3′-yl)-deoxyguanosine (E-3'-7-dG), 8-(trans-isoestragol-3′-yl)-deoxyguanosine (E-3'-8-dG), (Phillips et al., 1981; Punt et al., 2007) and N6 -(trans-isoestragol-3′-yl)-deoxyadenosine (E-3′-N 6 - dA) (Phillips et al., 1981). The major adduct formed with the guanine base is N2 -(trans-isoestragol-3′-yl)- deoxyguanosine (E-3′-N 2 -dG) which is considered to play a role in the genotoxic and carcinogenic effects induced by estragole (Philips et al., 1981; Smith et al, 2002). Recently it was reported that adducts between estragole and adenine (E-3′-N 6 -dA) were formed to a significant extent in the liver of male rats (F344) exposed to estragole at a dose level of 600 mg/kg bw for 4 weeks (Phillips et al., 1981; Ishii et al., 2011).


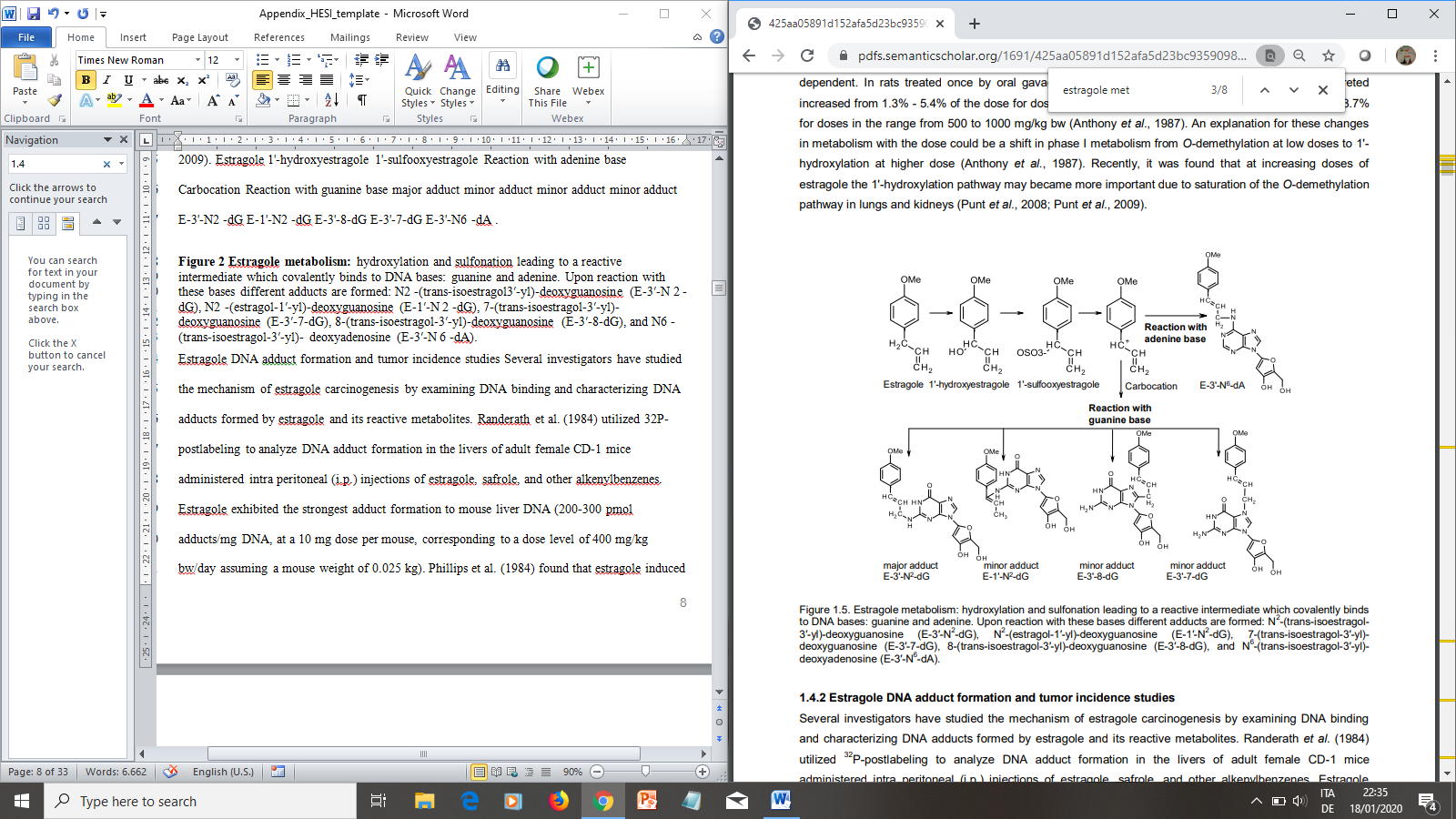


**Figure 2 Estragole metabolism:** hydroxylation and sulfonation leading to a reactive intermediate which covalently binds to DNA bases: guanine and adenine. Upon reaction with these bases different adducts are formed: N2 -(trans-isoestragol3′-yl)-deoxyguanosine (E-3′-N 2 -dG), N2 -(estragol-1′-yl)-deoxyguanosine (E-1′-N 2 -dG), 7-(trans-isoestragol-3′-yl)- deoxyguanosine (E-3′-7-dG), 8-(trans-isoestragol-3′-yl)-deoxyguanosine (E-3′-8-dG), and N6 -(trans-isoestragol-3′-yl)- deoxyadenosine (E-3′-N 6 -dA).

**Estragole DNA adduct formation and tumor incidence studies**: Several investigators have studied the mechanism of estragole carcinogenesis by examining DNA binding and characterizing DNA adducts formed by estragole and its reactive metabolites. Randerath et al. (1984) utilized ^32^P-postlabeling to analyze DNA adduct formation in the livers of adult female CD-1 mice administered intra peritoneal (i.p.) injections of estragole, safrole, and other alkenylbenzenes. Estragole exhibited the strongest adduct formation to mouse liver DNA (200-300 pmol adducts/mg DNA, at a 10 mg dose per mouse, corresponding to a dose level of 400 mg/kg bw/day assuming a mouse weight of 0.025 kg). Phillips et al. (1984) found that estragole induced adducts to liver DNA of newborn male B6C3F1 mice treated by i.p. injection on day 1, 8, 15 and 22 after birth at doses of 0.25, 0.5, 1.0 and 3.0 µmol per animal (with a final total dose of 4.75 µmol per animal). The DNA adduct levels observed in estragole-treated mice were 30.0, 14.8 and 9.4 pmol/mg DNA on days 23, 29 and 43, after birth respectively (Philips et al., 1984). Ishii et al. (2011) confirmed estragole-specific dG and dA adduct formation in vivo, using an isotope dilution LC-ESI-MS/MS method for measuring E-3′-N 6 -dA together with the two major dG adducts, E-3′-C 8 -dG and E3′-N 2 -dG. The levels of these adducts measured in the livers of F344 rats treated with estragole at a probably carcinogenic dose of 600 mg/kg bw for 4 weeks were respectively 3.5 E-3′-C 8 -dG adducts in 10^6^ dG, 4.8 E3′-N 2 -dG adducts in 106 dG, and 20.5 E-3′-N 6 -dA adducts in 10^6^ dA. Jeurissen et al. (2007) measured by LC-MS/MS the formation of 130 E-3′-N 2 -dG adducts in 10^6^ dG in HepG2 cells exposed to 50µM 1’-hydroxyestragole.

Tumor incidence studies are reported in Chapter 1 of Paini et al., but were not used to evaluate and validate the model (<https://pdfs.semanticscholar.org/1691/425aa05891d152afa5d23bc9359098569c8c.pdf>)

The present PBPK model is an extension of the PBPK models available to simulate estragole bioactivation and detoxification in rat (Punt et al., 2008) but also a human model was available (Punt et al., 2009) and was also extended (Punt et al., 2016). These PBPK models were defined based on literature data and in vitro metabolic parameters only, and provide possibilities to model metabolism of estragole at different oral doses in both species. These models provide more insight in relative dose- and species-dependent differences in bioactivation and detoxification of estragole, and are able to provide dose dependent predictions on the level of formation of the proximate and ultimate carcinogenic metabolites 1′ -hydroxyestragole and 1′ -sulfooxyestragole, respectively, in the target organ, the liver. In order to extend PBPK models to predict the overall toxic response and mode of action of a genotoxic compound should be described taking into account dynamics factors in addition to the kinetic factors. The PBPK model (Paini et al., 2010) was evaluated using literature data but additional validation and evaluation was performed using data from in vivo studies (Paini et al., 2012).

# 3. Model purpose

The purpose of development of this PBPK model was to develop new strategies for low dose cancer risk assessment of estragole by extending the PBPK models previously defined for simulating time concentration kinetics of estragole to predict DNA adduct formation. This will take the approach one step closer to the ultimate endpoint of tumor formation. Such models will facilitate risk assessment because they facilitate extrapolation from high to low dose levels, between species including human and between individuals. Furthermore, the PBPK model predicts in vivo DNA adduct formation based on only in vitro parameters contributes to the 3Rs (Replacement, Reduction and Refinement) for animal testing.

This PBPK model could be applied for risk assessment of genotoxic compounds, for instance it could be informative for the developed of EFSA Opinions on alkenylbenzene.

# 4. Materials and methods

PBPK models describe the body as a set of interconnected compartments, which represent the human organs and plasma, describing the absorption, distribution, metabolism, and excretion (ADME) characteristics of a compound within the body; by simulating the DNA adduct formation we include a description of the interaction of the chemical or its reactive metabolite with the toxicological receptor causing a first step towards the adverse effect.

### 4.1. Modeling strategy

The strategy adopted to develop the model was a six step approach for the development of a basic PBPK model illustrated by Rietjens et al., (2011), these steps include (see also figure 3):

1. definition of a conceptual model, which includes defining a simplified representation of the biological system and which model compartments can be included:
   1. Gather the biological knowledge and information on the ADME and MoA of estragole.
   2. The main target organ was found to be the liver as main organ where metabolism occurred, kidney and lungs were also checked but metabolism could be neglected.
   3. Establish the model structure (see 4.3)
2. translation of the conceptual model into a mathematical model by formulating a differential equation for each compartment;
   1. Establish the model equations (see 4.4)
3. defining the values of the parameters in the equations either from literature or from experiments:
   1. Gather the input parameter that were not measured 🡪 via literature search.
   2. Measuring in vitro the Vmax and Km of parent and metabolites.
   3. Synthesis the hydoxy metabolite/setup the analytics to measure the input parameters
   4. Expose of the rat primary hepatocyte to the metabolite to measure DNA adduct formation that then inform the model
4. solving the equations by calculating the concentrations of relevant compounds and their metabolites in the specific compartments;
   1. Write in Berkeley Madonna the equations
5. Validation and evaluation of the model performance with ultimate improvements to the model when needed;
   1. Mass balance was checked
   2. Sensitivity analysis was performed to check the sensitive parameters
   3. Validation using in vivo rodent data. SD in vivo test performed to see the amount formed of estragole DNA adduct in the liver to compare simulations with measured in vivo data.
   4. Refinement of the rat PBPK model from Punt et al. 2008 by adding liver zonation to better predict in vivo measurement as compared to the simulations
6. Making predictions by performing simulations.
   1. Application for food risk assessment of low dose genotoxic chemicals.


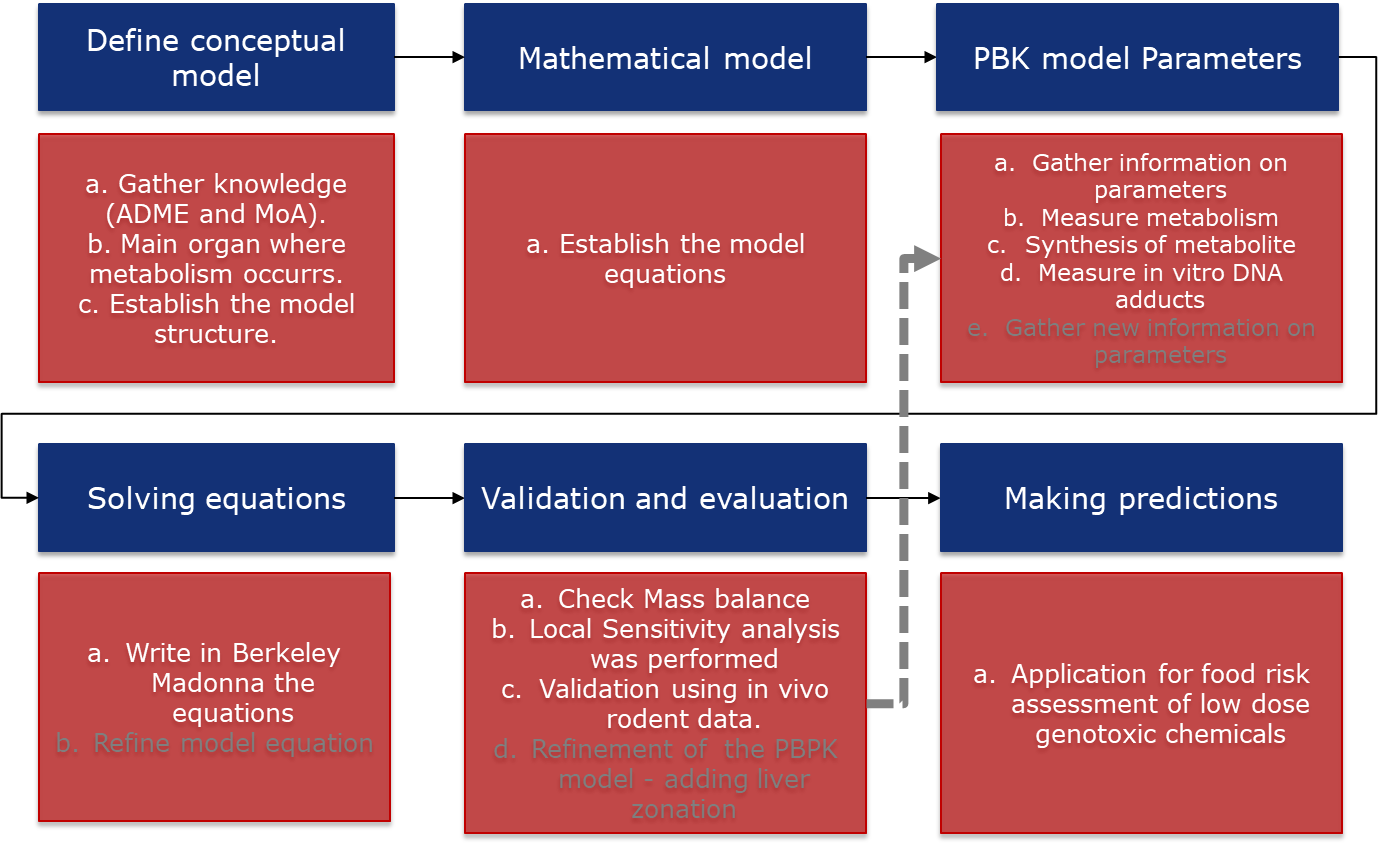


**Figure 3.** Schematic representation of the workflow used for model strategy to develop the PBPK model.

### 4.2. Summary of data for model development and evaluation

Information on biochemical (Vmax & Km) parameters measured using in vitro systems and time-dose concentration of DNA adduct formed and measured in vivo.

**Chemicals:**

1. Estragole was obtained from Acros Organics (Geel, Belgium). 2′-Deoxyguanosine was purchased from Sigma (Basel, Switzerland). 1,2,3,7,9-15N5-2′-Deoxyguanosine (15N5- dGuo) was obtained from Cambridge Isotope Laboratories (Cambridge, MA).
2. Synthesis of 1′-hydroxyestragole. 1′-Hydroxyestragole was synthesized based on a reaction described by Drinkwater et al. (1976) adapted from Borchert et al. (1973). (±)-1′-Hydroxyestragole (3.75 g [22.85 mmol, 80% isolated yield]) was isolated as a yellow oil. Satisfactory purity was demonstrated by NMR (N96%), HPLC (N96%), and GC-Tof analysis (pure).
3. Synthesis of 1′-acetoxyestragole. Purity of 1′-acetoxyestragole was verified by HPLC UV, NMR, and GCToF
4. Synthesis of E-3′-N2 -deoxyguanosine (E-3′-N2 -dGuo) and (15N5) E-3′- N2 -deoxyguanosine ((15N5) E-3′-N2 -dGuo). E-3′-N2 -dGuo and (15N5) E-3′-N2 -dGuo were prepared as described by Punt et al. (2007)

**Analytics:**

1. LC-ESI-MS/MS method for detection and quantification of E-3′-N2 -dGuo; 2.
2. Quantification of nucleoside E-3′-N2 -dGuo by isotope dilution.

**Cell Culture & exposure:**

1. Preparation and cultivation of primary rat hepatocytes. White male Sprague–Dawley rats, 8 weeks old, with a weight of 200–250 g, were used to isolate fresh primary hepatocytes by perfusion. Exposure of primary rat hepatocytes to 1′-hydroxyestragole. Rat primary hepatocytes were exposed to 1′-hydroxyestragole.
2. DNA extraction from primary hepatocytes and enzymatic digestion. DNA was extracted from primary rat hepatocytes exposed to the test compound using the Get pure DNA Kit-Cell protocol (Dojindo Molecular Technology Inc., Kumamoto, Japan) for 3 × 106 to 1 × 10^7^ cells (following the manufacturer's instructions).
3. Determination of 1′-hydroxyestragole in supernatant of primary rat hepatocytes
4. Cytotoxicity assay.

**In vivo study:** Animal Male Sprague Dawley rats, 6 weeks old, were used to perform a time and dose response study, information on the experimental set up can be found in Paini et al., (2012).

### 4.3. Model development and structure

Differential equations were integrated using the Berkley Madonna 8.0.1 software (Macey and Oster, UC Berkeley, CA, USA).

The PBPK model for rats was developed based on the PBPK model developed by Punt et al. (2008). The equation for DNA adduct formation was introduced into the PBPK rat model for estragole. In order to calculate the amount of DNA adducts formed at each external dose of estragole given, the model was extended as follows. The mass balance equation for the level of 1′-hydroxyestragole in the liver (L) reported in Punt et al. (2008) in the PBPK model was used to derive the AUC for 1′-hydroxyestragole in the liver within the first 2 h of exposure (Paini et al., 2010). To further improve the overall simulation of DNA adduct formation by the in vitro PPBK model, and to further reduce the differences between predicted and experimentally observed values, a refined PBPK model was defined taking two additional factors into account (Paini et al., 2012):

1. Liver zonation dividing the liver compartment within the PBPK model into three equal zone.
2. A submodel for 1'-OHES, to allow for entering the systemic circulation, and only 60% was allowed back into the liver.


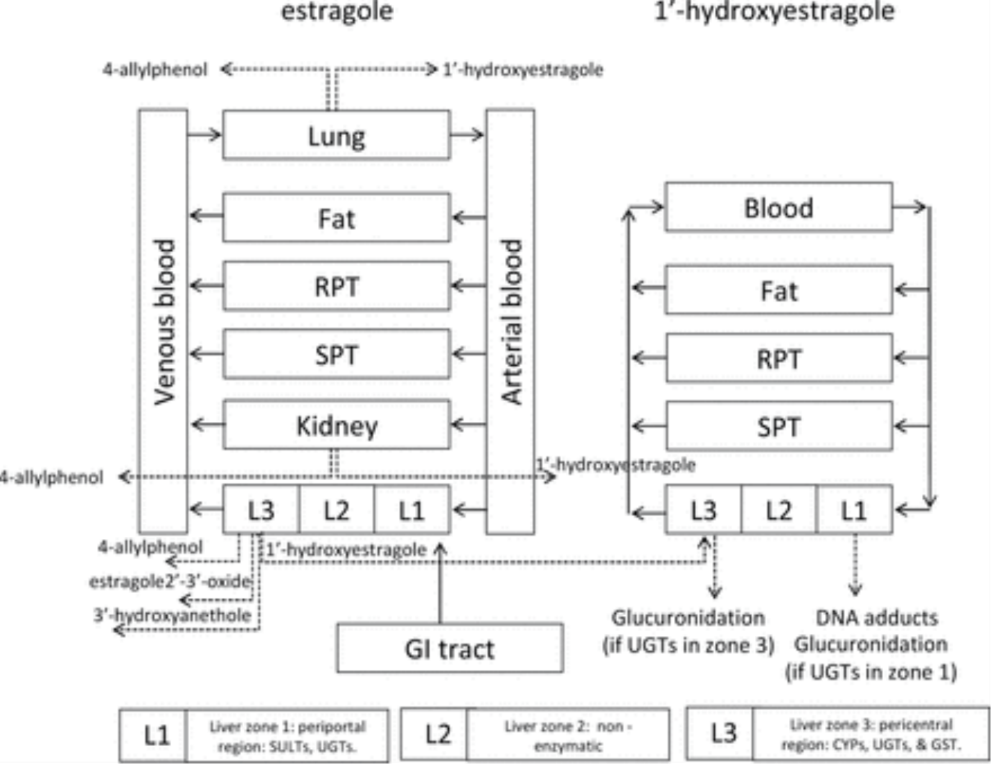


**Figure 4:** Schematic diagram of the new proposed PBPK model for estragole in rodents, with a 1'-OHES submodel and the liver compartment (L) subdivided into three equal zones: L1. A periportal zone containing the sulfotransferase (SULTs) activity and glucuronsyltransferases (UGTs). L2. A middle zone modelled without relevant enzyme activity. L3. A pericentral zone including the CYPs and glucuronsyltransferases (UGTs). GI, gastro intestinal tract; RPT, rapidly perfused tissue; SPT, slowly perfused tissue. (taken from Paini et al., 2012).

### 4.4. Model equations

Equations governing the rat PBPK model for estragole. (A= Amount, AM =amount metabolite)

**Uptake estragole from GI tract**

AGI = amount estragole remaining in GI tract, umol

dAGI /dt =-Ka*AGI

AGI = DOSE

**Slowly perfused tissue compartment**

AS = amount estragole in slowly perfused tissue, umol

dAS /dt = QS*(CA- ((AS/VS)/PSE))

**Richly perfused tissue compartment**

AR = amount estragole in richly perfused tissue, umol

dAR /dt = QR*(CA- ((AR/VR)/PRE))

**Fat compartment**

AF = amount estragole in fat tissue, umol

dAF /dt = QF*(CA- ((AF/VF)/PFE))

**Liver compartment (Zonation)**

AL1 = amount estragole in zone 1 of liver, umol

dAL1/dt = QL*(CA - (AL1/(VL/3))/PLE)+ Ka*AGI

AL2 = amount estragole in zone 2 of liver, umol

dAL2 /dt = QL*(CVL1 -((AL2/(VL/3))/PLE)

AL3 = amount estragole in zone 3 of liver, umol

dAL3 /dt = QL*(CVL2 -(AL3/(VL/3))/PL) - AMLHE /dt - AMLAP /dt - AMLEE /dt -AMLHA /dt

AMLHE = amount estragole metabolized in liver to 1’-hydroxyestragole (HE)

dAMLHE /dt = VmaxLHE*(AL3/(VL/3))/PL)/(KmLHE + (AL3/(VL/3))/PL)

AMLAP = amount estragole metabolized in liver to 4-allylphenol (AP)

dAMLAP /dt = VmaxLAP*(AL3/(VL/3))/PL)/(KmLAP + (AL3/(VL/3))/PL)

AMLEE = amount estragole metabolized in liver to estragole-2 /dt,3 /dt-oxide (EE)

dAMLEE /dt = VmaxLEE*(AL3/(VL/3))/PL)/(KmLEE + (AL3/(VL/3))/PL)

AMLHA = amount estragole metabolized in liver to 3 /dt-hydroxyanethole (HA)

dAMLHA /dt = VmaxLHA*(AL3/(VL/3))/PL)/(KmLHA + (AL3/(VL/3))/PL)

**Kidney compartment**

AK = amount estragole in kidney tissue, umol

dAK /dt = QK*(CA - (AK/VK)/PKE) - AMKHE /dt - AMKAP /dt

AMKHE = amount estragole metabolized in kidney to 1’-hydroxyestragole (HE)

dAMKHE /dt = VmaxKHE*((AK/VK)/PKE)/(KmKHE + (AK/VK)/PKE)

AMKAP = amount estragole metabolized in kidney to 4-allylphenol (AP)

dAMKAP /dt = VmaxKAP*((AK/VK)/PKE)/(KmKAP + (AK/VK)/PKE)

**Lung compartment**

ALu = amount estragole in lung tissue, umol

dALu /dt = QC*(CV-(ALu/VLu)/PLuE)) - AMLuHE /dt - AMLuAP /dt

AMLuHE = amount estragole metabolized in lung to 1’-hydroxyestragole (HE)

dAMLuHE /dt = VmaxLuHE*(ALu/VLu)/PLuE) /(KmLuHE + (ALu/VLu)/PLuE))

AMLuAP = amount estragole metabolized in lung to 4-allylphenol (AP)

dAMLuAP /dt = VmaxLuAP*(ALu/VLu)/PLuE) /(KmLuAP + (ALu/VLu)/PLuE))

**Arterial blood compartment**

AA = amount arterial blood estragole

dAA/dt = QC*(CALu- (AA/VA))

**Venous blood compartment**

AV = amount venous blood estragole (umol/L)

dAV /dt = (QF*((AF/VF)/PFE) + QR*((AR/VR)/PRE) + QS*- ((AS/VS)/PSE) + QL*(AL3/(VL/3))/PL)+ QK*(AK/VK)/PKE)) - QC*(AV/VV)

dAUCV /dt = AV/VV

**1’-hydroxyestragole submodel,** slowly perfused tissue compartment

ASHE = amount 1’-hydroxyestragole in slowly perfused tissue, umol

dASHE /dt = QS*(CAHE -ASHE/VS/PSHE)

1’-hydroxyestragole submodel, richly perfused tissue compartment

ARHE = amount 1’-hydroxyestragole in richly perfused tissue, umol

dARHE /dt = QR2*(CAHE -ARHE/VR2/PRHE)

1’-hydroxyestragole submodel, fat tissue compartment

AFHE = amount 1’-hydroxyestragole in fat tissue, umol

dAFHE /dt = QF*(CAHE -AFHE/VF/PFHE)

1’-hydroxyestragole submodel, liver tissue compartment (glucuronidation in **zone 1**)

AL1HE = amount 1’-hydroxyestragole in zone 1 of liver tissue, umol

dAL1HE /dt = QL*(CAHE*0.6-AL1HE/(VL/3) /PLHE)- AMLHES /dt - AMLHEG /dt

AL2HE = amount 1’-hydroxyestragole in zone 2 of liver tissue, umol

dAL2HE /dt = QL*(AL1HE/(VL/3) /PLHE -AL2HE/(VL/3)/PLHE)

AL3HE = amount 1’-hydroxyestragole in zone 3 of liver tissue, umol

dAL3HE /dt = AMLHE /dt +QL*(AL2HE/(VL/3)/PLHE-AL3HE/(VL/3)/PLHE) - AMLOE /dt

AMLHEG= amount 1’-hydroxyestragole metabolized in liver to 1’-hydroxyestragole glucurondie (HEG)

dAMLHEG /dt = VmaxLHEG*AL1HE/(VL/3) /PLHE/(KmLHEG + AL1HE/(VL/3) /PLHE)

CBWHEG = (AMLHEG/BW)*1000

AMLOE= amount 1’-hydroxyestragole metabolized in liver to 1 /dt-oxoestragole (OE)

dAMLOE /dt = VmaxLOE*AL3HE/(VL/3)/PLHE/(KmLOE + AL3HE/(VL/3)/PLHE)

AMLHES= amount 1’-hydroxyestragole metabolized in liver to 1 /dt-sulfooxyestragole (HES)

dAMLHES /dt = VmaxLHES*AL1HE/(VL/3) /PLHE/(KmLHES + AL1HE/(VL/3) /PLHE)

1’-hydroxyestragole submodel, liver tissue compartment (glucuronidation **in zone 3**)

AL1HE = amount 1’-hydroxyestragole in zone 1 of liver tissue, umol

dAL1HE /dt = QL*(CAHE*0.6-AL1HE/(VL/3) /PLHE)- AMLHES /dt

AL1HE/(VL/3) /PLHE= AL1HE/(VL/3) /PLHE

AL2HE = amount 1’-hydroxyestragole in zone 2 of liver tissue, umol

dAL2HE /dt = QL*(AL1HE/(VL/3) /PLHE -AL2HE/(VL/3)/PLHE)

AL2HE/(VL/3)/PLHE= AL2HE/(VL/3)/PLHE

AL3HE = amount 1’-hydroxyestragole in zone 3 of liver tissue, umol

dAL3HE /dt = AMLHE /dt +QL*(AL2HE/(VL/3)/PLHE-AL3HE/(VL/3)/PLHE) - dAMLOE /dt - dAMLHEG /dt

AL3HE/(VL/3)/PLHE= AL3HE/(VL/3)/PLHE

AMLHEG= amount 1‘-hydroxyestragole metabolized in liver to 1’-hydroxyestragole glucurondie (HEG)

dAMLHEG /dt = VmaxLHEG*AL3HE/(VL/3)/PLHE/(KmLHEG + AL3HE/(VL/3)/PLHE)

CBWHEG = (AMLHEG/BW)*1000

AMLOE= amount 1‘-hydroxyestragole metabolized in liver to 1 /dt-oxoestragole (OE)

dAMLOE /dt = VmaxLOE*AL3HE/(VL/3)/PLHE/(KmLOE + AL3HE/(VL/3)/PLHE)

AMLHES= amount 1’-hydroxyestragole metabolized in liver to 1 /dt-sulfooxyestragole (HES)

dAMLHES /dt = VmaxLHES*AL1HE/(VL/3) /PLHE/(KmLHES + AL1HE/(VL/3) /PLHE)

1‘-hydroxyestragole submodel, arterial and venous blood compartment

AVHE = amount venous blood 1’-hydroxyestragole (umol/L)

dAVHE/dt = (QR2*(ARHE/VR2/PRHE) + QF*(AFHE/VF/PFHE)+ QS*(ASHE/VS/PSHE)+ QL*(AL3HE/(VL/3)/PLHE)-QC* (AVHE/(VV+VA))

CVHE = AVHE/(VV+VA)

CAHE = CVHE

**DNA adduct formation**

dAUCLHE/dt = (AL1HE+AL2HE+AL3HE)/VL

The following equation was added to the model to describe the formation of E-3′-N2 -dGuo DNA adducts as a function of the AUC for 1′-hydroxyestragole:

DNAdGUO =A+B*AUCHE

The DNAdGUO (#adducts/1000 nt) is the amount of DNA adduct formed due to the specific binding of 1′-hydroxyestragole to deoxyguanosine nucleoside, A and B are the intercept and the slope calculated based on the data from the in vitro experiments.

### 4.5. Model parameters

The physiological parameters used in the estragole model were obtained from Brown et al. (1997). Partition coefficients were estimated from the log Kow based on a method of DeJongh et al. (1997) (Table 1). Log Kow values were estimated with the software package ClogP version 4.0 (Biobyte, Claremont, CA).

**Table 1.** Physiological Parameters and Partition Coefficients Used in the PBPK Model for Estragole in rats based on Brown et al., 1997 and DeJongh et al. (1997).

| Parameter | Abbreviation |  | | Value | Unit |
| --- | --- | --- | --- | --- | --- |
| Linear uptake | Ka |  | | 1 | hr-1 |
| Body weight | BW |  | | 0.263 (Stedev +/-10.25) | Kg |
|  | | | **Tissue volumes (% body weight)** | | |
| Liver | VL |  | | 3.4 |  |
| Lungs | VLu |  | | 0.5 |  |
| Kidneys | VK |  | | 0.7 |  |
| Fat | VF |  | | 7 |  |
| Rapidly perfused | VR |  | | 4.4 |  |
| Slowly perfused | VS |  | | 67.6 |  |
| Venous blood | VV |  | | 5.55 |  |
| Arterial blood | VA |  | | 1.85 |  |
| Rapidly perfused submodel | VR2 |  | | 9-VLc |  |
|  |  |  | |  |  |
| **Cardiac output** | CO |  | | 15 | **l/hr/kg bw0.74** |
|  | | | **Blood flow to tissue (% cardiac output)** | | |
| Liver | QL |  | | 25 |  |
| Kidneys | QK |  | | 14.1 |  |
| Fat | QF |  | | 7 |  |
| Rapidly perfused | QR |  | | 36.9 |  |
| Slowly perfused | QS |  | | 17 |  |
| Rapidly perfused submodel | QR2 |  | |  |  |
|  |  |  | |  |  |
|  | | | **Partition coefficients of estragole** | | |
| Liver:blood | PLE |  | | 2.4 |  |
| Lung:blood | PLuE |  | | 2.4 |  |
| Kidey:blood | PKE |  | | 2.4 |  |
| Fat:blood | PFE |  | | 76.9 |  |
| Rapidly perfused:blood | PRE |  | | 2.4 |  |
| Slowly perfused:blood | PSE |  | | 0.8 |  |
|  |  |  | |  |  |
|  | | | **Partition coefficient of 1-hydroxyestragole** | | |
| Liver:blood | PLHE |  | | 1.1 |  |
| Rapidly partition coefficient | PRHE |  | | 1.1 |  |
| Slowly partition coefficient | PSHE |  | | 0.55 |  |
| Fat partition coefficient | PFHE |  | | 9.92 |  |
|  |  |  | |  |  |
| **Metabolism Liver** |  |  | |  |  |
| metabolites of estragole |  |  | | unscaled maximum rate of metabolism |  |
| HE = 1'-hydroxyestragole, | VmaxLHEc |  | | 1.48 | (nmol min-1 (mg protein)-1) |
| AP = 4-allylphenol | VmaxLApc |  | | 0.85 | (nmol min-1 (mg protein)-1) |
| EE = estragole-2',3'-oxide | VmaxLEEc |  | | 2.16 | (nmol min-1 (mg protein)-1) |
| HA = 3'-hydroxyanethole | VmaxLHAc |  | | 1.05 | (nmol min-1 (mg protein)-1) |
| Affinity constants |  |  | |  |  |
| HE = 1'-hydroxyestragole, | KmLHE |  | | 116 | (umol/L) |
| AP = 4-allylphenol | KmLAP |  | | 458 | (umol/L) |
| EE = estragole-2',3'-oxide | KmLEE |  | | 154 | (umol/L) |
| HA = 3'-hydroxyanethole | KmLHA |  | | 93 | (umol/L) |
| metabolites of 1'-hydroxyestragole |  |  | | unscaled maximum rate of metabolism |  |
| HEG = 1'-hydroxyestragole glucuronide | KmLHEG |  | | 51.5 | (nmol min-1 (mg protein)-1) |
| OE= 1'-oxoestragole | KmLOE |  | | 2.9 | (nmol min-1 (mg protein)-1) |
| HES = 1'-sulfooxyestragole | KmLHES |  | | 0.019 | (nmol min-1 (mg protein)-1) |
| Affinity constants |  |  | |  |  |
| HEG = 1'-hydroxyestragole glucuronide | KmLHEG |  | | 203 | (umol/L) |
| OE= 1'-oxoestragole | KmLOE |  | | 1609 | (umol/L) |
| HES = 1'-sulfooxyestragole | KmLHES |  | | 63 | (umol/L) |
| **Metabolism Lung** |  |  | |  |  |
| metabolites of estragole |  |  | | unscaled maximum rate of metabolism |  |
| HE = 1'-hydroxyestragole | VmaxLuHEc |  | | 0.44 | (nmol min-1 (mg protein)-1) |
| AP = 4-allylphenol | VmaxLuAPc |  | | 0.67 | (nmol min-1 (mg protein)-1) |
| Affinity constants |  |  | |  |  |
| HE = 1'-hydroxyestragole | KmLuHE |  | | 25 | (umol/L) |
| AP = 4-allylphenol | KmLuAP |  | | 0.5 | (umol/L) |
| **Metabolism kidney** |  |  | |  |  |
| metabolites of estragole |  |  | | unscaled maximum rate of metabolism |  |
| HE = 1'-hydroxyestragole | VmaxKHEc |  | | 0.26 | (nmol min-1 (mg protein)-1) |
| AP = 4-allyphenol | VmaxKAPc |  | | 0.54 | (nmol min-1 (mg protein)-1) |
| Affinity constants |  |  | |  |  |
| HE = 1'-hydroxyestragole | KmKHE |  | | 22 | (umol/L) |
| AP = 4-allyphenol | KmKAP |  | | 0.5 | (umol/L) |
|  |  |  | |  |  |
| DNAadducts |  |  | | 0.032*AUCLHE |  |
|  | AUCLHE |  | | CLHE |  |

**Liver Scaling factors for metabolism**

S9PL=143 Liver S9 protein yield (mg/gram liver)

MPL=32 Liver microsomal protein yield (mg/gram liver)

L=VLC*1000 Liver volume (gram/kg BW)

metabolites of estragole, scaled maximum rate of metabolism (umol hr-1)

VMaxLHE = VMaxLHEc/1000*60*MPL*L*BW

VMaxLAP = VMaxLAPc/1000*60*MPL*L*BW

VMaxLEE = VMaxLEEc/1000*60*MPL*L*BW

VMaxLHA = VMaxLHAc/1000*60*MPL*L*BW

metabolites of 1'-hydroxyestragole, scaled maximum rate of metabolism (umol hr-1)

VMaxLHEG = VmaxLHEGc/1000*60*S9PL*L*BW

VMaxLOE = VmaxLOEc/1000*60*MPL*L*BW

VMaxLHES = VmaxLHESc/1000*60*S9PL*L*BW

**Lungs Scaling factors**

MPLu=20 ; Lung microsomal protein yield (mg/gram liver) Atio et al. 1976

Lu=VLuC*1000 ; Volume lung (gram/kg BW)

metabolites of estragole, scaled maximum rate of metabolism (umol hr-1)

VMaxLuHE = VMaxLuHEc/1000*60*MPLu*Lu*BW

VMaxLuAP = VMaxLuAPc/1000*60*MPLu*Lu*BW

**Kidney Scaling factors**

MPK=7 ; Kidney microsomal protein yield (mg/gram liver) Atio et al. 1976

K=VKC*1000 ; Volume kidney (gram/kg BW)

metabolites of estragole, unscaled maximum rate of metabolism (nmol min-1 (mg protein)-1)

metabolites of estragole, scaled maximum rate of metabolism (umol hr-1)

VMaxKHE = VMaxKHEc/1000*60*MPK*K*BW

VMaxKAP = VMaxKAPc/1000*60*MPK*K*BW

**DNA adduct data** established in vivo can be found Table 2. Results from the in vivo estragole excretion are also available in Table 2.

### 4.6. Model simulations

The PBPK model was run using oral route of exposure, to mimic the gavage. The exposure occurred as one bolus and simulations were run for 2hrs and 24 hrs. Exposure doses simulated using the SD rat PBPK model were 0.01, 0.07, 5, 30, 775, 150, 300 mg ES/kg BW.

### 4.7. Software

Differential equations were integrated using the Berkley Madonna 8.0.1 software (Macey and Oster, UC Berkeley, CA, USA), using Rosenbrock's algorithms for solving stiff systems. <https://berkeley-madonna.myshopify.com/>

The Berkeley Madonna User’s Guide - Version 8.0.2 - December 28, 2009 (<https://www.berkeleymadonna.com/system/storage/download/BM-Users-Guide-8.0.2.pdf>))

### Step-by-step instructions:

1. Enter (copy and paste) the model code reported in the appendix in the Berkley Madonna 8.0.1 software, Equation window;
2. Open the parameter window in order to be able to see all parameter and be able to change the values without touching the code;
3. Set the ODOSE to 0.01 or 0.07 mg/kg BW.
4. Run simulation: (check mass balance). Check time or dose dependent curves by using the graph window (a tabular format of the curve can be also exported).

# 5. Modeling results

### 5.1. Model evaluation

From the experimental data on the decrease of 1′-hydroxyestragole as a function of time in the incubations with rat primary hepatocytes exposed to 50 and 100 μM 1′-hydroxyestragole , the AUCHE(in vitro) values were calculated. These experimental data obtained at 50 and 100 μM 1′-hydroxyestragole were also presented as the decrease in the percentage of the original concentration of the test compound. From these experimental results obtained at 50 and 100 μM of 1′- hydroxyestragole, the time-dependent decrease in the concentration of 1′-hydroxyestragole at other starting concentrations of 1′-hydroxyestragole was calculated and the theoretical data thus obtained to generate theoretical curves allowed calculation of the area under time curve (AUCHE(in vitro)) values for the whole range of 1′-hydroxyestragole concentrations. In a next step, these AUCHE(in vitro) values were used to replace the x-axis values, resulting in representation of the level of E-3′-N2 -dGuo DNA adduct formation in rat primary hepatocytes as a function of the AUC for 1′-hydroxyestragole (AUCHE(in vitro)), Figure 5. This reveals a linear relationship with an intercept at the origin. Thus, the curve presented can be fitted by a linear equation passing through zero according to:

DNAdGUO = 0.0324*AUCHE with an r^2^= 0.9635,

and DNAdGUO representing the amount of E-3′-N2 - dGuo DNA adducts (adducts/1000 nt) formed in the hepatocytes at a certain AUCHE(in vitro) (h×μmol/l) of 1′-hydroxyestragole. By incorporating this equation into the PBPK model and defining that AUCHE(in vitro) should equal AUCHE (h ×μmol/l) in the in vivo PBPK model defined by Punt et al. (2008) a link between the AUC predicted in the PBPK model (AUCHE) and the AUC from the hepatocyte incubations (AUCHE(in vitro)) was established; ultimately providing a link between the PBK model and the formula for DNA binding in vitro and defining a PBPK model that can predict DNA binding as a function of estragole dose. Figure 6 presents a comparison between the dose-dependent formation of estragole–DNA adducts in the liver of male Sprague Dawley rats as experimentally determined in this study and as predicted by the PBPK model. The results reveal that the PBPK model–based predictions closely match the experimentally observed values. The PBPK model predictions at 48h upon dosing appear to slightly overestimate DNA adduct formation by a factor of 1.9–2.3-fold. Figure 7 shows in addition the simulations of DNA adducts formed and glucuronidation of estragole run with the refined PBPK model including liver zonation.


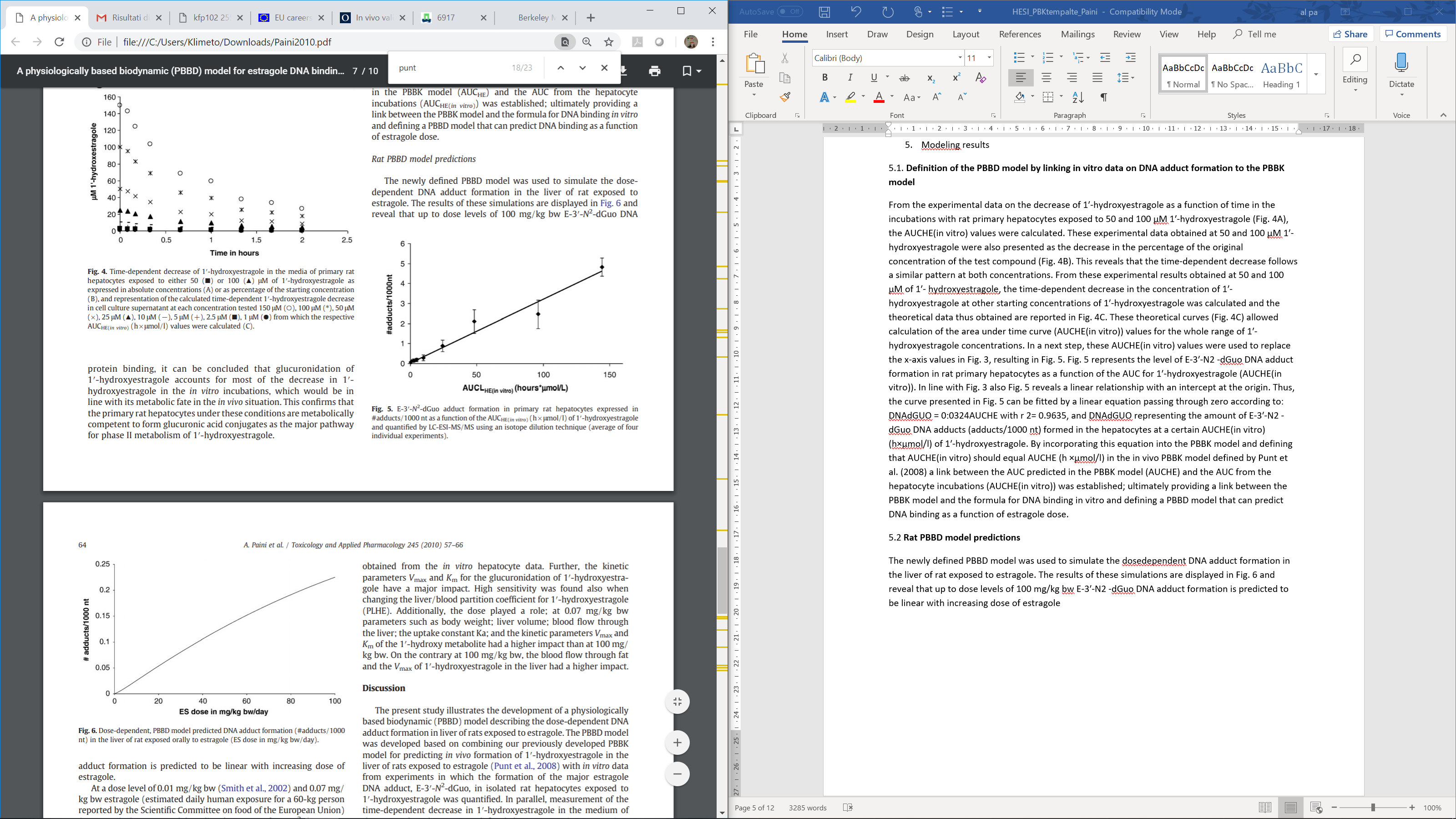


**Figure 5**. E-3′-N^2^-dGuo [adduct](https://www.sciencedirect.com/topics/earth-and-planetary-sciences/adduct) formation in primary rat hepatocytes expressed in #adducts/1000 nt as a function of the AUC_HE(in vitro)_ (h × μmol/l) of 1′-hydroxyestragole and quantified by LC-ESI-MS/MS using an [isotope](https://www.sciencedirect.com/topics/pharmacology-toxicology-and-pharmaceutical-science/isotope) dilution technique (average of four individual experiments).


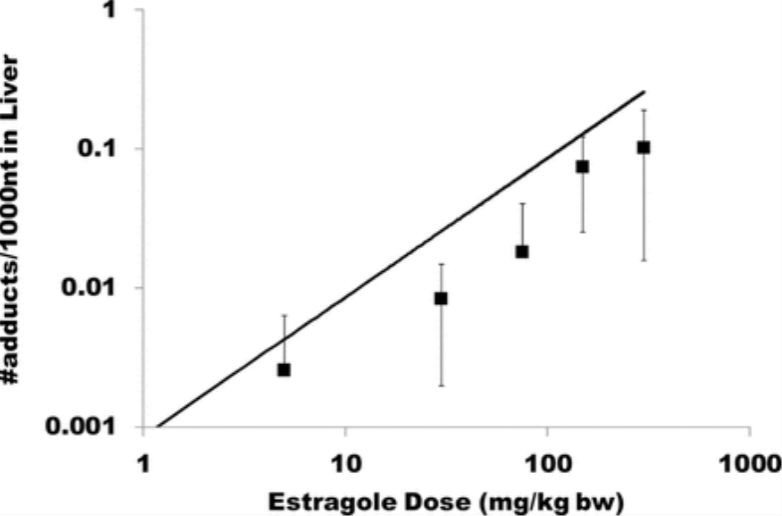


**Figure 6**: In vitro PBPK model (solid line) prediction of the DNA adduct formation at 48h versus DNA adducts measured in vivo (black square) at different oral doses of estragole. Data represent the average and standard deviation of 10 rats.


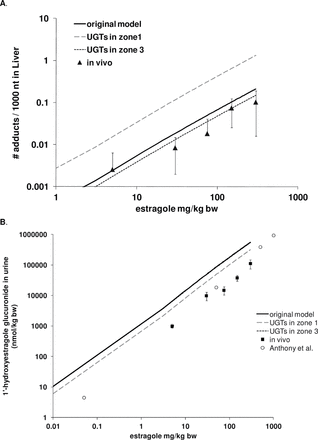


**Figure 7A**. Comparison of experimental data obtained in this study for DNA adduct formation in the liver to their PBK/PBPK−based predicted values when using either the original model (black lines) or the newly defined extended PBK/PBPK models including liver zonation and a submodel for 1'-hydroxyestragole; (dashed lines). The two zonal models include UGTs in either Zone 3 (dashed black line) or Zone 1 (dashed grey line) and in vivo experimental data are presented as black triangles and represent the average and standard deviation of 10 rats. 8B. Comparison of experimental data obtained in this study for formation of urinary 1'-hydroxyestragole glucuronide to their PBK/PBPK−based predicted values when using either the original model (black lines) or the newly defined extended PBK/PBPK models including liver zonation and a submodel for 1'-hydroxyestragole; (dashed lines). The two zonal models include UGTs in either Zone 3 (dashed black line) or Zone 1 (dashed grey line). Data presented as black squares are the results of this study whereas those presented as open circles are those reported by Anthony et al. (5) for female Wistar Albino rats. Urinary data (black squares) represent the average and standard deviation of eight rats.

**Table 2**. Individual rat data for estragole DNA adduct (#adduct/1000 nt) formation and urinary excretion of the 1’-OHES (nmol/kgbw) as glucuronide conjugate.

### 5.2. Sensitivity, uncertainty, and variability analyses

Normalized sensitivity coefficients (SC) were determined to identify parameters that largely influence the prediction of the maximal blood concentrations (Cmax) in liver blood by the PBPK model, using the equation:

SC = ((C’-C)/(P’-P))*(P/C)

where P and Pʹ are the initial and modified parameter values respectively, and C and Cʹ are the initial and modified values of the model output resulting from an increase in parameter value, respectively (Evans and Andersen, 2000). A 5% increase in parameter value was chosen to analyze the effect of a change in a parameter. Local, one at time, sensitivity analysis was performed at different estragole doses to identify the key parameters that can influence the DNA adduct formation in the PBPK model outcome. The estragole doses set for sensitivity analysis were 0.07 mg/kg bw (Figure 8, corresponding to the average daily intake for humans, SCF, 2001) and 100 mg/kg bw (the highest dose at which the model prediction is linear).


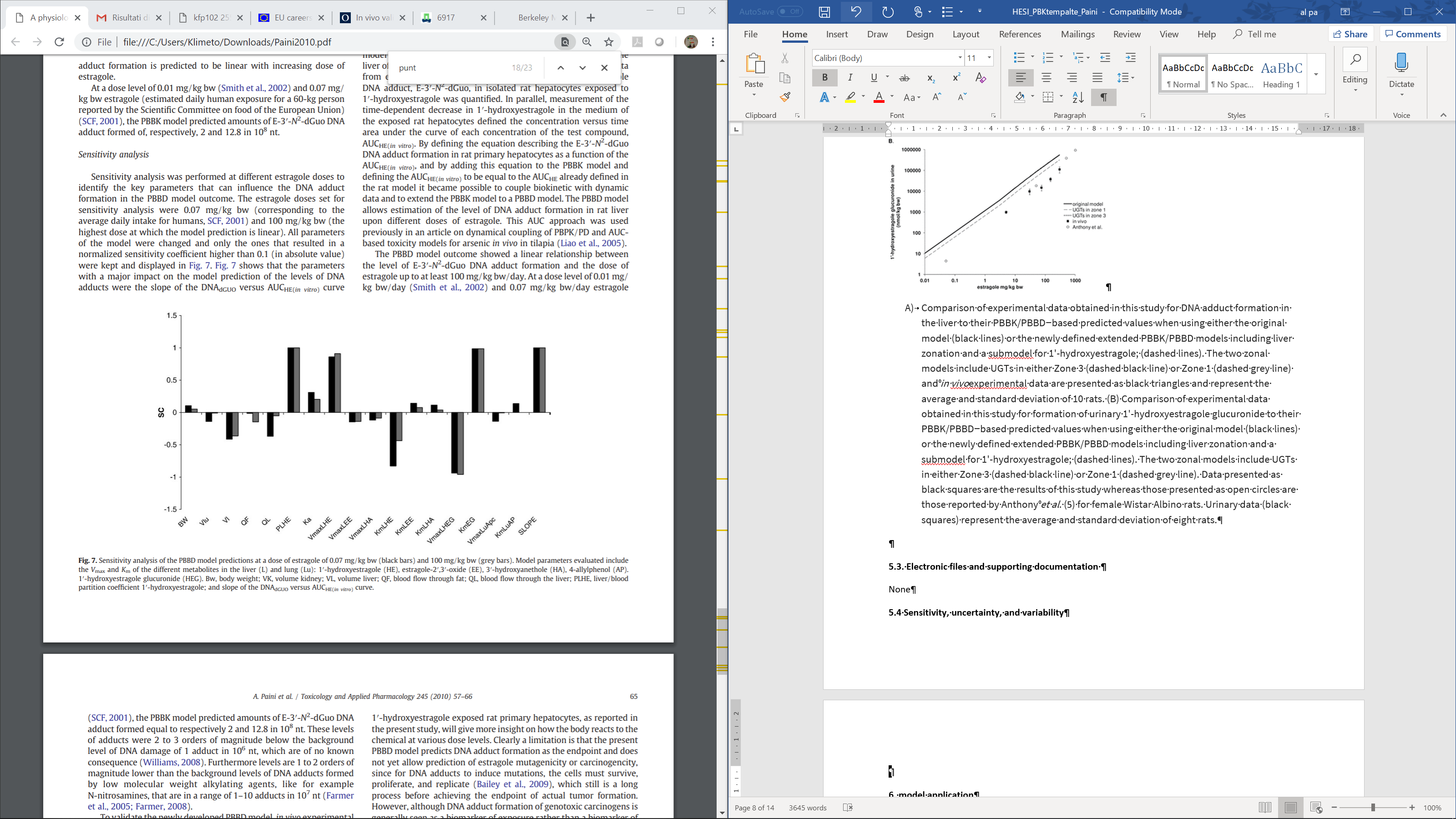


**Figure 8**. Sensitivity analysis of the PBPK model predictions at a dose of [estragole](https://www.sciencedirect.com/topics/pharmacology-toxicology-and-pharmaceutical-science/estragole) of 0.07 mg/kg bw (black bars) and 100 mg/kg bw (grey bars). Model parameters evaluated include the V_max_ and K_m_ of the different metabolites in the liver (L) and lung (Lu): 1′-hydroxyestragole (HE), estragole-2′,3′-oxide (EE), 3′-hydroxyanethole (HA), 4-allylphenol (AP). 1′-hydroxyestragole [glucuronide](https://www.sciencedirect.com/topics/pharmacology-toxicology-and-pharmaceutical-science/glucuronide) (HEG). Bw, body weight; VK, volume kidney; VL, volume liver; QF, blood flow through fat; QL, blood flow through the liver; PLHE, liver/blood [partition coefficient](https://www.sciencedirect.com/topics/earth-and-planetary-sciences/partition-coefficient) 1′-hydroxyestragole; and slope of the DNA_dGUO_ versus AUC_HE(in vitro)_ curve.

|  |  | **UNCERTAINTY** | | |
| --- | --- | --- | --- | --- |
|  |  | High | Medium | Low |
| **SENSITIVITY** | High | PLHE  High sensitivity was found also when changing the liver/blood [partition coefficient](https://www.sciencedirect.com/topics/earth-and-planetary-sciences/partition-coefficient) for 1′-hydroxyestragole (PLHE). Uncertainty is also high since the PC is based on a QSAR (Brown et al., 1997) and not on measured data. | VmaxLHEG & KmEG – VmaxLHE  Metabolic parameters were measured and have a high SA on model output and could have a medium uncertainty due to the fact that there can be human errors and analytical limits in measuring. | SLOPE  The in PBPK model to predict DNA adducts was first built using in vitro measurements. But was then checked in vivo and resulted in (+/-) 2 fold from in vivo data. Uncertainty is low while the SA is high due to the high impact this will have on the DNA adduct prediction of the model |
|  | Medium | Ka  Uptake constant has a medium impact on model outcome based on the SA but has high uncertainty due to the fact that was assumed a liner uptake of the chemical, without considering real absorption. |  | VL  The volume of the liver will have a medium impact on the model output based on SA. The value cannot differ so much so uncertainty is low. |
|  | Low |  |  |  |

**Figure 9.** Evaluation of model parameters sensitivity versus uncertainty based on results of the SA reported in figure 9. SA = Sensitivity analysis.

Sensitivity analysis was performed at different estragole doses to identify the key parameters that can influence the DNA adduct formation in the PBPK model outcome. The estragole doses set for sensitivity analysis were 0.07 mg/kg bw (corresponding to the average daily intake for humans, [SCF, 2001](https://www.sciencedirect.com/science/article/pii/S0041008X10000414?via%3Dihub#bib31)) and 100 mg/kg bw (the highest dose at which the model prediction is linear). All parameters of the model were changed and only the ones that resulted in a normalized sensitivity coefficient higher than 0.1 (in absolute value) were kept and displayed in Figure 9. The results show that the parameters with a major impact on the model prediction of the levels of DNA adducts were the slope of the DNA_dGUO_ versus AUC_HE(in vitro)_ curve obtained from the in vitro hepatocyte data. Further, the kinetic parameters V_max_ and K_m_ for the glucuronidation of 1′-hydroxyestragole have a major impact. High sensitivity was found also when changing the liver/blood [partition coefficient](https://www.sciencedirect.com/topics/earth-and-planetary-sciences/partition-coefficient) for 1′-hydroxyestragole (PLHE). Additionally, the dose played a role; at 0.07 mg/kg bw parameters such as body weight; liver volume; blood flow through the liver; the uptake constant Ka; and the kinetic parameters V_max_ and K_m_ of the 1′-hydroxy metabolite had a higher impact than at 100 mg/kg bw. On the contrary, at 100 mg/kg bw, the blood flow through fat and the V_max_ of 1′-hydroxyestragole in the liver had a higher impact.

### 5.3. Model applicability

The newly defined PBPK model was used to simulate the dose dependent DNA adduct formation in the liver of rat exposed to estragole. The results of these simulations reveal that up to dose levels of 100 mg/kg bw E-3′-N2 -dGuo DNA adduct formation is predicted to be linear with increasing dose of estragole.

At a dose level of 0.01 mg/kg bw (Smith et al., 2002) and 0.07 mg/ kg bw estragole (estimated daily human exposure for a 60-kg person reported by the Scientific Committee on food of the European Union) (SCF, 2001), the PBK model predicted amounts of E-3′-N2 -dGuo DNA adduct formed of, respectively, 2 and 12.8 in 108 nt.

This PBPK model can be applied for risk assessment of genotoxic compounds, for instance it could be informative for the developed of EFSA Opinions on alkenylbenzene.

# 6. Discussion and conclusions

The purpose of development of this PBPK model was to develop new strategies for low dose cancer risk assessment of estragole by extending the PBPK models previously defined for simulating time concentration kinetics of estragole to predict DNA adduct formation. This will take the approach one step closer to the ultimate endpoint of tumor formation. Such models will facilitate risk assessment because they facilitate extrapolation from high to low dose levels, between species including human and between individuals. Furthermore, the PBPK model predicts in vivo DNA adduct formation based on only in vitro parameters contributes to the 3Rs (Replacement, Reduction and Refinement) for animal testing.

The present PBPK model is an extension of the PBPK models available to simulate estragole bioactivation and detoxification in rat (Punt et al., 2008) but also a human model was available (Punt et al., 2009) and was also extended (Punt et al., 2016). These PBPK models were defined based on literature data and in vitro metabolic parameters only, and provide possibilities to model metabolism of estragole at different oral doses in both species. These models provide more insight in relative dose- and species-dependent differences in bioactivation and detoxification of estragole, and are able to provide dose dependent predictions on the level of formation of the proximate and ultimate carcinogenic metabolites 1′ -hydroxyestragole and 1′ -sulfooxyestragole, respectively, in the target organ, the liver. In order to extend PBPK models to predict the overall toxic response and mode of action of a genotoxic compound should be described taking into account dynamics factors in addition to the kinetic factors. The PBPK model (Paini et al., 2010) was evaluated using literature data but additional validation and evaluation was performed using data from in vivo studies (Paini et al., 2012).

# 7. Electronic files and supporting documents

### Estragole rat PBPK model Model Code

The model was already published previously by Punt et al., [Toxicol Appl Pharmacol. 2008 Sep 1;231(2):248-59. Epub 2008 Apr 27.(16)] and Paini et al., [[Toxicol Appl Pharmacol.](http://www.ncbi.nlm.nih.gov/pubmed/20144636) 2010 May 15;245(1):57-66. Epub 2010 Feb 6. (15)]. Equations describing the liver zonation and submodel for the estragole metabolite 1’-hydroxyestragole are included in the model code Paini et al., 2010, 2012. Equations are solved with the Berkeley Madonna Software (Copyright ^©^1993-2001 Robert I. Macey & George F. Oster - <http://www.berkeleymadonna.com/>).

;===========================================================================

;Physiological parameters

;==========================================================================

;Tissue volumes

BW = 0.263 {Kg} ; body weight rat

VLc = 0.043 ; fraction of liver tissue

VKc = 0.006 ; fraction of kidney tissue

VLuc = 0.0053 ; fraction of lung tissue

VFc = 0.07 ; fraction of fat tissue

VAc = 0.0185 ; fraction of arterial blood: 0.074*1/4

VVc = 0.0555 ; fraction of venous blood: 0.074*3/4

VRc = 0.09-VLc-VLuc-VKc ; fraction of richly perfused tissue

VSc = 0.82-VFc-VAc-VVc ; fraction of blood flow to slowly perfused tissue

VR2c = 0.09-VLc ; fraction of richly perfused tissue in 1’-hydroxyestragole submodel

VL = VLc*BW

VLu= VLuc*BW

VK = VKc*BW

VF = VFc*BW

VR = VRc*BW

VS = VSc*BW

VA = VAc*BW

VV = VVc*BW

VR2 = VR2c*BW

;-------------------------------------------------------------------------------------------------;Blood flow rates

QC = 15*BW**0.74 {L/hr}

QLc = 0.25 ; fraction of blood flow to liver

QKc = 0.141 ; fraction of blood flow to kidney

QFc = 0.07 ; fraction of blood flow to fat

QRc = 0.76-QLC-QKC ; fraction of blood flow to richly perfused tissue

QSc = 0.24-QFC ; fraction of blood flow to slowly perfused tissue

QR2c = 0.76-QLC ; fraction of blood flow to richly perfused tissue in 1’-hydroxyestragole submodel

QL = QLc*QC

QK = QKc*QC

QF = QFc*QC

QR = QRc*QC

QS = QSc*QC

QR2 =QR2c*QC

;=========================================================================

;Partition Coefficients

;========================================================================

;estragole

PLE = 2.4 ; liver/blood partition coefficient

PLuE=2.4 ; lung/blood partition coefficient

PKE=2.4 ; kidney/blood partition coefficient

PFE = 76.9 ; fat/blood partition coefficient

PRE = 2.4 ; richly perfused tissues/blood partition coefficient

PSE = 0.8 ; slowly perfused tissues/blood partition coefficient

;1'-hydroxyestragole

PLHE = 1.1 ;liver/blood partition coefficient

PRHE = 1.1 ;rapidly partitio coefficient

PSHE = 0.55 ;slowly partition coefficient

PFHE = 9.92 ;fat partition coefficient

;==============================================================

;Biochemical parameters

;==============================================================

;Linear uptake rate (hr-1)

Ka = 1

;---------------------------------------------------------------;Metabolism liver

;Scaling factors

S9PL=143; Liver S9 protein yield (mg/gram liver)

MPL=32; Liver microsomal protein yield (mg/gram liver)

L=VLC*1000; Liver volume (gram/kg BW)

;metabolites of estragole, unscaled maximum rate of metabolism (nmol min-1 (mg protein)-1)

VmaxLHEc = 1.48 ;HE = 1'-hydroxyestragole,

VmaxLAPc = 0.85 ;AP = 4-allylphenol

VmaxLEEc = 2.16 ;EE = estragole-2',3'-oxide

VmaxLHAc = 1.05 ;HA = 3'-hydroxyanethole

;metabolites of estragole, scaled maximum rate of metabolism (umol hr-1)

VMaxLHE = VMaxLHEc/1000*60*MPL*L*BW

VMaxLAP = VMaxLAPc/1000*60*MPL*L*BW

VMaxLEE = VMaxLEEc/1000*60*MPL*L*BW

VMaxLHA = VMaxLHAc/1000*60*MPL*L*BW

;metabolites of estragole, affinity constants (umol/L)

KmLHE = 116

KmLAP = 458

KmLEE = 154

KmLHA = 93

;metabolites of 1'-hydroxyestragole, unscaled maximum rate of metabolism (nmol min-1 (mg protein)-1)

VmaxLHEGc = 51.5 ; HEG = 1'-hydroxyestragole glucuronide

VmaxLOEc = 2.9 ; OE= 1'-oxoestragole

VmaxLHESc = 0.019 ; HES = 1'-sulfooxyestragole

;metabolites of 1'-hydroxyestragole, scaled maximum rate of metabolism (umol hr-1)

VMaxLHEG = VmaxLHEGc/1000*60*S9PL*L*BW

VMaxLOE = VmaxLOEc/1000*60*MPL*L*BW

VMaxLHES = VmaxLHESc/1000*60*S9PL*L*BW

;metabolites of 1'-hydroxyestragole, affinity constants (umol/L)

KmLHEG = 203

KmLOE = 1609

KmLHES = 63

;----------------------------------------------------------------------------------- ------------------------;Metabolism lung

;Scaling factors

MPLu=20 ; Lung microsomal protein yield (mg/gram liver) Atio et al. 1976

Lu=VLuC*1000 ; Volume lung (gram/kg BW)

;metabolites of estragole, unscaled maximum rate of metabolism (nmol min-1 (mg protein)-1)

VmaxLuHEc = 0.44 ; HE = 1'-hydroxyestragole

VmaxLuAPc = 0.67 ; AP = 4-allylphenol

;metabolites of estragole, scaled maximum rate of metabolism (umol hr-1)

VMaxLuHE = VMaxLuHEc/1000*60*MPLu*Lu*BW

VMaxLuAP = VMaxLuAPc/1000*60*MPLu*Lu*BW

;metabolites of estragole, affinity constants (umol/L)

KmLuHE = 25

KmLuAP = 0.5

;-----------------------------------------------------------------------------------------------;Metabolism kidney

;Scaling factors

MPK=7 ; Kidney microsomal protein yield (mg/gram liver) Atio et al. 1976

K=VKC*1000 ; Volume kidney (gram/kg BW)

;metabolites of estragole, unscaled maximum rate of metabolism (nmol min-1 (mg protein)-1)

VmaxKHEc = 0.26 ; HE = 1'-hydroxyestragole

VmaxKAPc = 0.54 ; AP = 4-allyphenol

;metabolites of estragole, scaled maximum rate of metabolism (umol hr-1)

VMaxKHE = VMaxKHEc/1000*60*MPK*K*BW

VMaxKAP = VMaxKAPc/1000*60*MPK*K*BW

;metabolites of estragole, affinity constants (umol/L)

KmKHE = 22

KmKAP = 0.5

;==========================================================================

;Run settings

;==========================================================================

;Molecular weight

MWE = 148.2; Molecular weight estragole

MWHE=164.2; Molecular weight 1'-hydroxyestragole

;Given dose (mg/ kg bw) and oral dose umol/ kg bw}

GDOSE = 0.07 {mg/ kg bw} ; GDOSE = given dose

ODOSE = GDOSE*1E-3/MWE*1E6 {umol/ kg bw} ; ODOSE = given dose recalculated to umol/kg bw

DOSE=ODOSE*BW; ; DOSE = umol

;Time

Starttime = 0; in hrs

Stoptime = 48; in hrs

;=====================================

;Dynamics

;=====================================

;slowly perfused tissue compartment

;AS = amount estragole in slowly perfused tissue, umol

AS' = QS*(CA-CVS)

Init AS = 0

CS = AS/VS

CVS = CS/PSE

;--------------------------------------------------------;richly perfused tissue compartment

;AR = amount estragole in richly perfused tissue, umol

AR' = QR*(CA-CVR)

Init AR = 0

CR = AR/VR

CVR = CR/PRE

;-------------------------------------------------;fat compartment

;AF = amount estragole in fat tissue, umol

AF' = QF*(CA-CVF)

Init AF = 0

CF = AF/VF

CVF = CF/PFE

;---------------------------------------------------------;uptake estragole from GI tract

;AGI = amount estragole remaining in GI tract,umol

AGI' =-Ka*AGI

Init AGI = DOSE

;------------------------------------------------------------;liver compartment

;AL1 = amount estragole in zone 1 of liver, umol

AL1' = QL*(CA -CVL1)+ Ka*AGI

Init AL1 = 0

CL1 = AL1/(VL/3)

CVL1=CL1/PLE

;AL2 = amount estragole in zone 2 of liver, umol

AL2' = QL*(CVL1 -CVL2)

Init AL2 = 0

CL2 = AL2/(VL/3)

CVL2=CL2/PLE

;AL3 = amount estragole in zone 3 of liver, umol

AL3' = QL*(CVL2 -CVL3) - AMLHE' - AMLAP' - AMLEE' -AMLHA'

Init AL3 = 0

CL3 = AL3/(VL/3)

CVL3=CL3/PL

;AMLHE = amount estragole metabolized in liver to 1'-hydroxyestragole (HE)

AMLHE' = VmaxLHE*CVL3/(KmLHE + CVL3)

init AMLHE = 0

;AMLAP = amount estragole metabolized in liver to 4-allylphenol (AP)

AMLAP' = VmaxLAP*CVL3/(KmLAP + CVL3)

init AMLAP = 0

;AMLEE = amount estragole metabolized in liver to estragole-2',3'-oxide (EE)

AMLEE' = VmaxLEE*CVL3/(KmLEE + CVL3)

init AMLEE = 0

;AMLHA = amount estragole metabolized in liver to 3'-hydroxyanethole (HA)

AMLHA' = VmaxLHA*CVL3/(KmLHA + CVL3)

init AMLHA = 0

;-----------------------------------------------------------------------------------

;AK = amount estragole in kidney tissue, umol

AK' = QK*(CA -CVK) - AMKHE' - AMKAP'

Init AK = 0

CK = AK/VK

CVK = CK/PKE

;AMKHE = amount estragole metabolized in kidney to 1'-hydroxyestragole (HE)

AMKHE' = VmaxKHE*CVK/(KmKHE + CVK)

init AMKHE = 0

;AMKAP = amount estragole metabolized in kidney to 4-allylphenol (AP)

AMKAP' = VmaxKAP*CVK/(KmKAP + CVK)

init AMKAP = 0

;------------------------------------------------------------------ ;lung compartment

;ALu = amount estragole in lung tissue, umol

ALu' = QC*(CV-CALu) - AMLuHE' - AMLuAP'

Init ALu = 0

CLu = ALu/VLu

CALu = CLu/PLuE

;AMLuHE = amount estragole metabolized in lung to 1'-hydroxyestragole (HE)

AMLuHE' = VmaxLuHE*CALu/(KmLuHE + CALu)

init AMLuHE = 0

;AMLuAP = amount estragole metabolized in lung to 4-allylphenol (AP)

AMLuAP' = VmaxLuAP*CALu/(KmLuAP + CALu)

init AMLuAP = 0

;-------------------------------------------------------------------------------; arterial blood compartment

;AA = amount arterial blood estragole

AA' = QC*(CALu-CA);

Init AA = 0

CA = AA/VA

;-----------------------------------------------------------------------------; venous blood compartment

;AV = amount venous blood estragole (umol/L)

AV' = (QF*CVF + QR*CVR + QS*CVS + QL*CVL3+ QK*CVK) - QC*CV

Init AV = 0

CV = AV/VV

AUCV' = CV

init AUCV = 0

;----------------------------------------;1’-hydroxyestragole submodel, slowly perfused tissue compartment

;ASHE = amount 1’-hydroxyestragole in slowly perfused tissue, umol

ASHE' = QS*(CAHE -CVSHE)

Init ASHE = 0

CSHE = ASHE/VS

CVSHE = CSHE/PSHE

;--------------------------------------;1’-hydroxyestragole submodel, richly perfused tissue compartment

;ARHE = amount 1’-hydroxyestragole in richly perfused tissue, umol

ARHE' = QR2*(CAHE -CVRHE)

Init ARHE = 0

CRHE = ARHE/VR2

CVRHE = CRHE/PRHE

;------------------------------------------------;1’-hydroxyestragole submodel, fat tissue compartment

;AFHE = amount 1’-hydroxyestragole in fat tissue, umol

AFHE' = QF*(CAHE -CVFHE)

Init AFHE = 0

CFHE = AFHE/VF

CVFHE = CFHE/PFHE

;------------------;1’-hydroxyestragole submodel, liver tissue compartment (glucuronidation in zone 1)

;AL1HE = amount 1’-hydroxyestragole in zone 1 of liver tissue, umol

AL1HE' = QL*(CAHE*0.6-CVL1HE)- AMLHES' - AMLHEG'

Init AL1HE = 0

CL1HE = AL1HE/(VL/3)

CVL1HE=CL1HE/PLHE

;AL2HE = amount 1’-hydroxyestragole in zone 2 of liver tissue, umol

AL2HE' = QL*(CVL1HE -CVL2HE)

Init AL2HE = 0

CL2HE = AL2HE/(VL/3)

CVL2HE=CL2HE/PLHE

;AL3HE = amount 1’-hydroxyestragole in zone 3 of liver tissue, umol

AL3HE' = AMLHE' +QL*(CVL2HE-CVL3HE) - AMLOE'

Init AL3HE = 0

CL3HE = AL3HE/(VL/3)

CVL3HE=CL3HE/PLHE

;AMLHEG= amount 1'-hydroxyestragole metabolized in liver to 1'-hydroxyestragole glucurondie (HEG)

AMLHEG' = VmaxLHEG*CVL1HE/(KmLHEG + CVL1HE)

init AMLHEG = 0

CBWHEG = (AMLHEG/BW)*1000

;AMLOE= amount 1'-hydroxyestragole metabolized in liver to 1'-oxoestragole (OE)

AMLOE' = VmaxLOE*CVL3HE/(KmLOE + CVL3HE)

init AMLOE = 0

;AMLHES= amount 1'-hydroxyestragole metabolized in liver to 1'-sulfooxyestragole (HES)

AMLHES' = VmaxLHES*CVL1HE/(KmLHES + CVL1HE)

init AMLHES = 0

;----------------;1’-hydroxyestragole submodel, liver tissue compartment (glucuronidation in zone 3)

;AL1HE = amount 1’-hydroxyestragole in zone 1 of liver tissue, umol

;AL1HE' = QL*(CAHE*0.6-CVL1HE)- AMLHES'

;Init AL1HE = 0

;CL1HE = AL1HE/(VL/3)

;CVL1HE=CL1HE/PLHE

;AL2HE = amount 1’-hydroxyestragole in zone 2 of liver tissue, umol

;AL2HE' = QL*(CVL1HE -CVL2HE)

;Init AL2HE = 0

;CL2HE = AL2HE/(VL/3)

;CVL2HE=CL2HE/PLHE

;AL3HE = amount 1’-hydroxyestragole in zone 3 of liver tissue, umol

;AL3HE' = AMLHE' +QL*(CVL2HE-CVL3HE) - AMLOE' - AMLHEG'

;Init AL3HE = 0

;CL3HE = AL3HE/(VL/3)

;CVL3HE=CL3HE/PLHE

;AMLHEG= amount 1'-hydroxyestragole metabolized in liver to 1'-hydroxyestragole glucurondie (HEG)

;AMLHEG' = VmaxLHEG*CVL3HE/(KmLHEG + CVL3HE)

;init AMLHEG = 0

;CBWHEG = (AMLHEG/BW)*1000

;AMLOE= amount 1'-hydroxyestragole metabolized in liver to 1'-oxoestragole (OE)

;AMLOE' = VmaxLOE*CVL3HE/(KmLOE + CVL3HE)

;init AMLOE = 0

;AMLHES= amount 1'-hydroxyestragole metabolized in liver to 1'-sulfooxyestragole (HES)

;AMLHES' = VmaxLHES*CVL1HE/(KmLHES + CVL1HE)

;init AMLHES = 0

;------------------------------------; ;1’-hydroxyestragole submodel, arterial and venous blood compartment

;AVHE = amount venous blood 1’-hydroxyestragole (umol/L)

AVHE' = (QR2*CVRHE + QF*CVFHE+QS*CVSHE+QL*CVL3HE)-QC*CVHE;

Init AVHE = 0

CVHE = AVHE/(VV+VA)

CAHE = CVHE

;===========================================================================

;Mass balance calculations

;===========================================================================

{Mass Balance}

Total = DOSE

Calculated = AF + AS + AR + AL1+AL2+AL3 + AK+ ALu + AV+ AA + AGI + AMLEE + AMLHA+ AMLHE + AMLAP + AMKHE + AMKAP + AMLuHE + AMLuAP

ERROR=((Total-Calculated)/Total+1E-30)*100

MASSBBAL=Total-Calculated + 1

;============================================================================

;Calculations with model

;============================================================================

;DNA adduct formation - link to dynamics

CLHE = (AL1HE+AL2HE+AL3HE)/VL

AUCLHE'= CLHE

Init AUCLHE=0

DNAdGuo=0.032*AUCLHE

# 8. Appendices

### Abbreviations

15N5-dG 1,2,3,7,9-15N5-2′-Deoxyguanosine (15N5-dG)

BMD Benchmark Dose

BMR Benchmark Response

dG 2'-deoxyguanosine

E-1'-N2-dA N6-(trans-isoestragol-3′-yl)-deoxyadenosine

E-3'-7-dG 7-(trans-isoestragol-3′-yl)-deoxyguanosine

E-1'-N2-dG N 2-(estragol-1′-yl)-deoxyguanosine

E-3'-8-dG 8-(trans-isoestragol-3′-yl)-deoxyguanosine

E-3'-N2-dG N 2-(trans-isoestragol-3′-yl)-deoxyguanosine

EDI Estimated Daily Intake

EFSA European Food Safety Authority

ES Estragole

FEMA Flavor and Extract Manufacturers Association

GC-MS Gas Chromatography – Mass Spectrometry

GSH Glutathione

GST Glutathione-s-transferase

HE 1'-Hydroxyestragole

HEG 1'-Hydroxyestragole glucuronide

HPLC High Performance Liquid Chromatography

JECFA Joint FAO/WHO Expert Committee on Food Additives

LC-MS Liquid Chromatography – Mass Spectrometry

MOA Mode of Action

MOE Margin of Exposure

nt nucleotide

OE 1′-oxoestragole

PBPK Physiologically based pharmacokinetic

PFS Plant Food Supplements

SCF-EU Scientific Committee on Food of the European Union

SD Sprague Dawley

Stdev Standard Deviation

SULT Sulfotransferase

VSD Virtual Safe Dose

### References

Abdo KM, Cunningham ML, Snell ML, Herbert RA, Travlos GS, Eldridge SR, and Bucher JR. (2001) 14-week toxicity and cell proliferation of methyleugenol administered by gavage to F344 rats and B6C3F1 mice. Food and Chemical Toxicology 39: 303-316

Anthony A. Caldwell J. Hutt A. J. Smith R. L. 1987 Metabolism of estragole in rat and mouse and influence of dose size on excretion of the proximate carcinogen 1′-hydroxyestragole. Food Chem. Toxicol., 25 799 806.

Bock, K.W., Brunner, G., Hoensch, H., Huber, E., Josting, D., 1978. Determination ofmicrosomal UDP-glucuronyltransferase in needle-biopsy specimens of humanliver. Eur. J. Clin. Pharmacol. 14, 367–373

Brown R.P., Delp M.D., Lindstedt S.L., Rhomberg L.R., Beliles R.P. Physiological parameter values for physiologically based pharmacokinetic models Toxicol. Ind. Health, 13 (1997), pp. 407-484

DeJongh J., Verhaar H.J.M., Hermens J.L.M. A quantitative property-property relationship (QPPR) approach to estimate in vitro tissue-blood partition coefficients of organic chemicals in rats and humans. Arch. Toxicol., 72 (1997), pp. 17-25

Ishii Y. Suzuki Y. Hibi D. Jin M. Fukuhara K. Umemura T. Nishikawa A 2011 Detection and quantification of specific DNA adducts by liquid chromatography−tandem mass spectrometry in the livers of rats given estragole at the carcinogenic dose. Chem. Res. Toxicol., 24, 532 541.

Iyer, L.V., Ho, M.N., Shinn, W.M., Bradford, W.W., Tanga, M.J., Nath, S.S., Green, C.E.,2003. Glucuronidation of 1-hydroxyestragole (1′-HE) by Human UDP-Glucurono-syltransferases UGT2B7 and UGT1A9. Toxicol. Sci. 73, 36–43

Jeurissen S. M. F. Punt A. Boersma M. G. Bogaards J. J. Fiamegos Y. C. Schilter B. van Bladeren P. J. Cnubben N. H. P. Rietjens I. M. C. M. 2007 Human cytochrome P450 enzyme specificity for the bioactivation of estragole and related alkenylbenzenes. Chem. Res. Toxicol., 20 798 806.

Miller, E.C., Swanson, A.B., Phillips, D.H., Fletcher, T.L., Liem, A., Miller, J.A., 1983.Structure–activity studies of the carcinogenicities in the mouse and rat of somenaturally occurring and synthetic alkenylbenzene derivatives related to safrole andestragole. Cancer Res. 43, 1124–1134.

Paini A, Punt A, Viton F, Scholz G, Delatour T, Marin-Kuan M, Schilter B, van Bladeren PJ, Rietjens IM. (2010) A physiologically based biodynamic (PBPK) model for estragole DNA binding in rat liver based on in vitro kinetic data and estragole DNA adduct formation in primary hepatocytes. Toxicol Appl Pharmacol. 2010 May 15;245(1):57-66. doi: 10.1016/j.taap.2010.01.016. Epub 2010 Feb 6.

Paini A.,Punt A., Scholz G, Gremaud E., Spenkelink B., Alink G, Schilter B, van Bladeren PJ , I.M.C.M. Rietjens (2012) In vivo validation of DNA adduct formation by estragole in rats predicted by physiologically based biodynamic modelling, Mutagenesis, Volume 27, Issue 6, 1 November 2012, Pages 653–663, https://doi.org/10.1093/mutage/ges031

Phillips D. H. Reddy M. V. Randerath K. 1984 P-32 Post-labeling analysis of DNA adducts formed in the livers of animals treated with safrole, estragole and other naturally-occurring alkenylbenzenes. 2. Newborn male B6c3f1 mice. Carcinogenesis, 5 1623 1628

Phillips, D.H., 2005. DNA adducts as markers of exposure and risk. Mutation Res. 577(1-2), 284–292.

Phillips, D.H., Miller, J.A., Miller, E.C., Adams, B., 1981. Structures of the DNA adductsformed in mouse liver after administration of the proximate hepatocarcinogen1′-hydroxyestragole. Cancer Res. 41, 176–186.

Punt A., Paini A., Spenkelink A., Scholz G., Schilter B., van Bladeren P.J., Rietjens I.M. Evaluation of interindividual human variation in bioactivation and DNA adduct formation of estragole in liver predicted by physiologically based kinetic/dynamic (PBK/D) and Monte Carlo modelling. Chem. Res. Toxicol. 2016;29:659–668.

Punt, A., Delatour, T., Scholz, G., Schilter, B., van Bladeren, P.J., Rietjens, I.M.C.M., 2007.Tamdem mass spectrometry analysis of N2-(trans-isoestragole-3′-yl)deoxyguano-sine as a strategy to study species differences in sulfotransferase conversion of theproximate carcinogen 1′-hydroxyestragole. Chem. Res. Tox. 20 (7), 991–998.

Punt, A., Freidig, A.P., Delatour, T., Scholz, G., Schilter, B., Boersma, M.G., van Bladeren, P.J., Rietjens, I.M.C.M., 2008. A physiologically based biokinetic (PBK) model for estragole bioactivation and detoxification in rat. Toxicol. Appl. Pharmacol. 231 (2), 248–259.

Punt, A., Paini, A., Boersma, M.G., Freidig, A.P., Delatour, T., Scholz, G., Schilter, B., vanBladeren,P.J., Rietjens, I.M.C.M., 2009. Use of physiologically based biokinetic(PBBK) modeling to study estragole bioactivation and detoxification in humans ascompared to male rats. Toxicol. Sci. 110 (2), 255–269

Rietjens IMCM, Louisse J, and Punt A. (2011) Tutorial on physiologically based kinetic modelling in molecular nutrition and food research. Molecular Nutrition & Food Research 55(6): 941–956.

SCF (Scientific Committee on Food) (2001), Opinion of the scientific committee on food on estragole (1-allyl-4-methoxybenzene),http://ec.europa.eu/food/fs/sc/scf/out104_en.pdf.

Smith, R.L., Adams, T.B., Doull, J., Feron, V.J., Goodman, J.I., Marnett, L.J., Portoghese, P.S.,Waddell, W.J., Wagner, B.M., Rogers, A.E., Caldwell, J., Sipes, I.G., 2002. Safetyassessment of allylalkoxybenzene derivatives used asflavouring substances—methyl eugenol and estragole. Food Chem. Toxicol. 40, 851–870.

Swanson AB, Miller EC, and Miller JA. (1981) The Side-Chain Epoxidation and Hydroxylation of the Hepatocarcinogens Safrole and Estragole and Some Related-Compounds by Rat and Mouse-Liver Microsomes. Biochimica Et Biophysica Acta 673, 504-516.

Van den Berg SJPL, Restani P, Boersma MG, Delmulle L, and Rietjens IMCM. (2011) Levels of genotoxic and carcinogenic ingredients in plant food supplements and associated risk assessment Food and Nutrition Sciences, Food and Nutrition Sciences 2, 989-1010
